# The CERV protein of Cer1, a *C*. *elegans* LTR retrotransposon, is required for nuclear export of viral genomic RNA and can form giant nuclear rods

**DOI:** 10.1371/journal.pgen.1010804

**Published:** 2023-06-29

**Authors:** Bing Sun, Haram Kim, Craig C. Mello, James R. Priess

**Affiliations:** 1 RNA Therapeutics Institute, University of Massachusetts Medical School, Worcester,United States of America; 2 Program in Molecular Medicine, University of Massachusetts Medical School, Worcester, United States of America; 3 Howard Hughes Medical Institute, Chevy Chase, Maryland, United States of America; 4 Fred Hutchinson Cancer Center, Seattle, Washington, United States of America; University of California San Diego, UNITED STATES

## Abstract

Retroviruses and closely related LTR retrotransposons export full-length, unspliced genomic RNA (gRNA) for packaging into virions and to serve as the mRNA encoding GAG and POL polyproteins. Because gRNA often includes splice acceptor and donor sequences used to splice viral mRNAs, retroelements must overcome host mechanisms that retain intron-containing RNAs in the nucleus. Here we examine gRNA expression in Cer1, an LTR retrotransposon in *C*. *elegans* which somehow avoids silencing and is highly expressed in germ cells. Newly exported Cer1 gRNA associates rapidly with the Cer1 GAG protein, which has structural similarity with retroviral GAG proteins. gRNA export requires CERV (*C*. *e**legans* regulator of viral expression), a novel protein encoded by a spliced Cer1 mRNA. CERV phosphorylation at S214 is essential for gRNA export, and phosphorylated CERV colocalizes with nuclear gRNA at presumptive sites of transcription. By electron microscopy, tagged CERV proteins surround clusters of distinct, linear fibrils that likely represent gRNA molecules. Single fibrils, or groups of aligned fibrils, also localize near nuclear pores. During the *C*. *elegans* self-fertile period, when hermaphrodites fertilize oocytes with their own sperm, CERV concentrates in two nuclear foci that are coincident with gRNA. However, as hermaphrodites cease self-fertilization, and can only produce cross-progeny, CERV undergoes a remarkable transition to form giant nuclear rods or cylinders that can be up to 5 microns in length. We propose a novel mechanism of rod formation, in which stage-specific changes in the nucleolus induce CERV to localize to the nucleolar periphery in flattened streaks of protein and gRNA; these streaks then roll up into cylinders. The rods are a widespread feature of Cer1 in wild strains of *C*. *elegans*, but their function is not known and might be limited to cross-progeny. We speculate that the adaptive strategy Cer1 uses for the identical self-progeny of a host hermaphrodite might differ for heterozygous cross-progeny sired by males. For example, mating introduces male chromosomes which can have different, or no, Cer1 elements.

## Author summary

LTR retrotransposons are closely related to retroviruses and are enormously abundant in animals and plants. Cer1 is the most prevalent LTR retrotransposon in the nematode *C*. *elegans*, where it is expressed at high levels in adult germ cells. Cer1 produces typical retroviral proteins except for a novel protein called CERV. CERV appears to allow unspliced Cer1 genomic RNA to be exported from the nucleus, escaping host mechanisms that normally retain unspliced RNA. The nuclear export of gRNA is regulated by multiple ON/OFF switches controlled by sex, developmental stage, and environment. CERV is a diffuse nucleoplasmic protein in the OFF states, but in the ON states CERV colocalizes with nuclear gRNA. By transmission electron microscopy, CERV is associated with unusual and distinct linear fibrils that appear to represent gRNA molecules. In older germ cells, CERV undergoes a remarkable transition to form giant cylindrical rods of unknown function that can equal the nuclear diameter in length. Rod formation occurs when *C*. *elegans* hermaphrodites can no longer fertilize their own oocytes, and instead require mating with males. Thus, the rods might be part of adaptive strategies Cer1 uses to distinguish potentially heterozygous cross-progeny from homozygous self-progeny.

## Introduction

Long terminal repeat (LTR) retrotransposons are ubiquitous in animals and plants; they comprise about 8% of the human genome and over 70% of plant genomes such as maize and wheat [1,2]. LTR retrotransposons appear to contribute to human aging and to chronic diseases such as autoimmune disorders [3–7], and are pathogens in several plants [8,9]. Conversely, LTR retrotransposons can have positive roles; some elements have been co-opted for host functions such as viral defense, and LTR retrotransposons have had major impacts on the evolution of gene regulatory networks and genome variation [10–13].

LTR retrotransposons are closely related to retroviruses but lack Envelope (Env) proteins involved in horizontal transmission. Retroviruses such as Rous Sarcoma Virus (RSV) and Murine Leukemia Virus (MLV) have a simple genomic organization in the form 5’ LTR-*gag*-*pol*-*env*-3’ LTR. GAG and POL are the products of a large, unspliced mRNA that can double as the viral genomic RNA (gRNA), while ENV is the product of a separate, spliced mRNA [14]. GAG is the major structural component of the virion and forms the protein shell or capsid for the viral genome. The GAG polyprotein has three major domains: The nucleocapsid (NC) domain contains one or more Cys-Cys-His-Cys (CCHC) zinc fingers that bind viral gRNA, the capsid (CA) domain multimerizes to form the immature viral particles, and matrix (MA) typically targets the developing particles to the plasma membrane [14]. The POL (polymerase) polyprotein is proteolytically cleaved into smaller, conserved enzymes such as Protease, Reverse Transcriptase, RNase H, and Integrase. Retroviral ENV proteins generally are class I membrane fusion proteins, although the ENV protein of the hookworm Atlas retrovirus is a structurally unrelated class II membrane protein which resembles the *C*. *elegans* cell-cell fusogen EFF-1/AFF-1 [15]. Endogenous retroviruses often lose *env* genes as they adapt to their hosts, and some LTR retrotransposons appear to acquire *env* genes de novo [16,17]. Many LTR retrotransposons, particularly in plants, contain an open reading frame (ORF) in the expected position for an *env* gene, but in most cases this ORF has no clear homology and its function is unknown [18].

The genomes of *Caenorhabditis elegans* wild strains contain several families of LTR retrotransposons and a few retroviruses, collectively called Cer elements (*C*. *e**legans* retrotransposon) [19–22]. The most prevalent element is Cer1, a member of the Gypsy/Ty3 family of retroviruses/retrotransposons [19,21]. Cer1 has inserted at multiple sites over all chromosomes in wild populations of *C*. *elegans*; for example, Cer1 LTRs occur at 182 unique sites in 208 sequenced strains [20]. The laboratory N2 strain of *C*. *elegans* contains multiple remnants of extinct Cer1 elements plus a single, potentially intact element, Cer1[N2, LGIII:-0.04], which inactivates the mucin-like gene *plg-1*; this insertion appears to be recent and is the basis for copulatory plug dimorphism in wild strains [21,23,24]. Viral-like particles of Cer1 GAG are highly abundant in adult hermaphrodite gonads, where they are visible by transmission electron microscopy (TEM) and by immunostaining [24]. Cer1 GAG particles initially escaped experimental detection because most laboratory culture of *C*. *elegans* is at 20–22°C, while GAG is expressed preferentially at 15°C and not detected at 25°C [24]. The GAG particles appear to bind and bundle gonad microtubules, and contribute to cytoskeletal defects and temperature-dependent infertility in aging adults [24]. These observations raise the question of how or why Cer1 expression is tolerated, given that *C*. *elegans* has robust small RNA-mediated silencing pathways that repress DNA transposons and other types of Cer retroelements [25–29]. Recent studies suggest that Cer1 has been co-opted by *C*. *elegans* to transfer learned memories of pathogen avoidance, both horizontally and transgenerationally; although the mechanism is unclear, the transfer appears to involve Cer1 GAG particles and information that is somehow relayed to neurons [30]. Interestingly, the mammalian neuronal protein Arc is involved in long-term memory and appears to be derived from a GAG protein [31–33]; Arc forms viral-like particles that can transfer horizontally as extracellular vesicles [34–37].

Our present understanding of Cer1 protein functions is largely inferred by sequence comparisons with retroviruses and other LTR retrotransposons. The predicted Cer1 POL polyprotein has clear similarity to the enzymatic domains of retroviral POL proteins, such as reverse transcriptase and integrase, and these domains occur in the same 5’ to 3’ order characteristic of the Gypsy/Ty3 family of retroviruses [19,21]. The predicted Cer1 GAG polyprotein contains an NC domain with three CCHC fingers, but lacks sequence similarity to the MA or CA domains of retroviral GAG polyproteins [21]. Cer1 has a large open reading frame (ORF) between *pol* and the 3’LTR in the expected position for a retroviral ENV protein [19]. However, the Cer1 ORF does not resemble retroviral ENV proteins or the fusogen EFF-1/AFF-1, and has no obvious homology with proteins outside of *Caenorhabditis* [24]. The ORF forms the C-terminal half of a larger protein which is encoded by a spliced mRNA [24]; based on the findings in the present study, we designate this protein as CERV (*C*. *e**legans* regulator of viral RNA).

Here we focus on characterizing the GAG and CERV proteins, which are the most species-specific components of Cer1. Using structural modeling we identify a domain in Cer1 GAG which closely resembles known structures for retroviral CA domains and is predicted to have a similar ability to multimerize. We demonstrate that a large fraction of GAG particles in germ cells are associated with gRNA, and that these particles appear to protect Cer1 gRNA from natural, age-dependent degradation and from experimentally imposed RNAi-mediated degradation. We show that CERV is a nuclear protein which is not required for gRNA transcription but is essential for gRNA export from the nucleus to the cytoplasm. gRNA is exported in sex, stage, and region-specific conditions where CERV concentrates at nuclear foci of gRNA, the presumptive sites of Cer1 transcription. Conversely, gRNA is not exported under different conditions where CERV does not concentrate on nuclear gRNA. This ON/OFF switch in export is coincident with, and dependent on, phosphorylation of CERV at residue S214. Transmission electron microscopy of germ nuclei shows that CERV is concentrated around clusters of small, linear fibrils which likely correspond to gRNA molecules; other CERV-associated fibrils are aligned in linear arrays suggestive of export intermediates. Most remarkably, we show that CERV can form giant, cylindrical nuclear rods, at least some of which contain gRNA. The rods occur primarily after hermaphrodites have finished producing self-progeny, but retain the ability to produce cross-progeny when mated with males. The CERV rods occur in various wild strains of *C*. *elegans* with different Cer1 insertion sites, suggesting they are a general but mysterious addition to our understanding of Cer1 biology.

## Results

### Background

**Fig 1:**
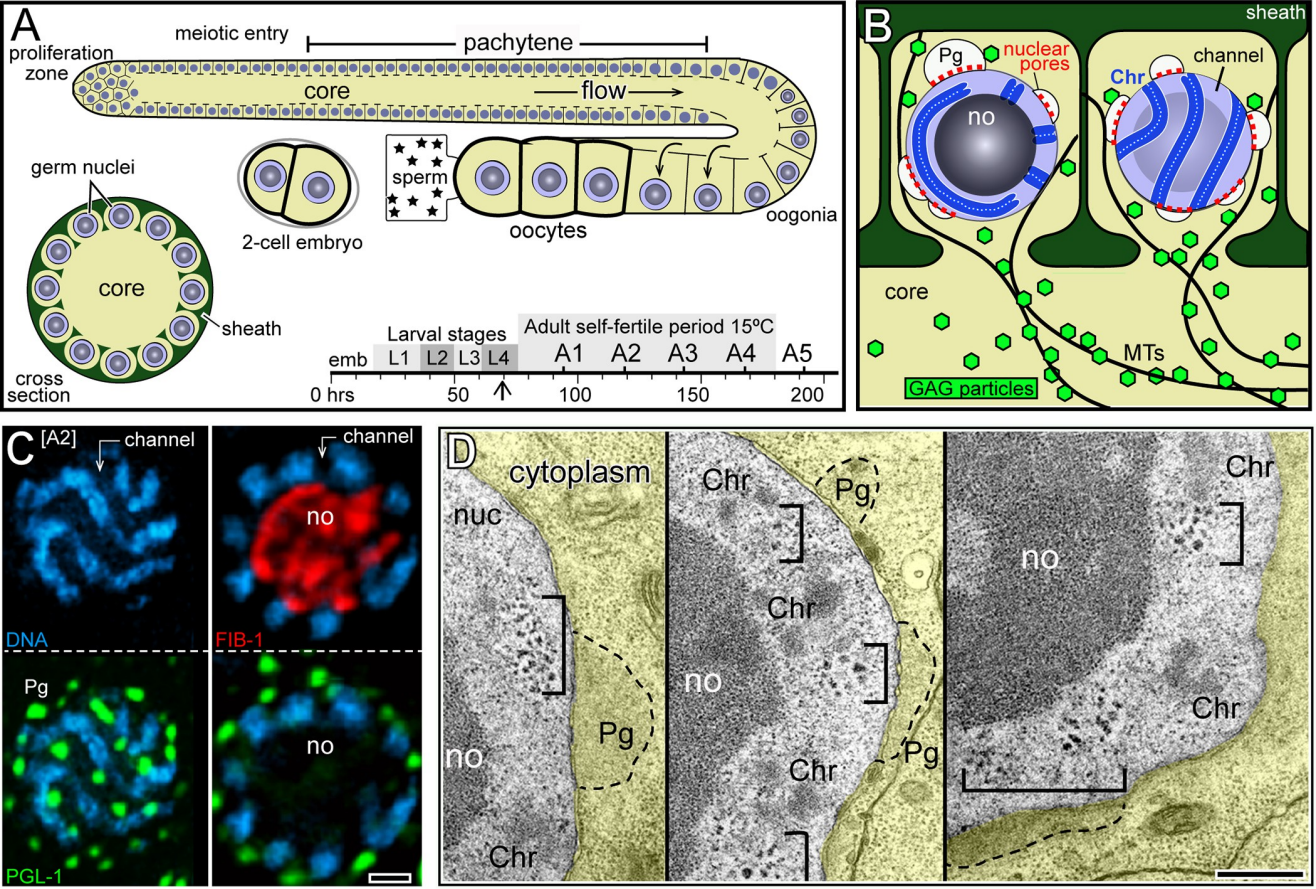
*C*. *elegans* gonad and pachytene germ cells. **A.** Diagrams representing longitudinal (top) and cross-sectional (bottom left) views of one of the two arms of the adult hermaphrodite gonad. Germ cells are covered by peripheral somatic cells called sheath cells (green). The distal end of the gonad contains proliferating germ cells, and cells exiting the proliferation zone enter meiotic prophase. Hermaphrodite germ cells initially differentiate as sperm which are stored, but all later germ cells differentiate as oocytes. The hermaphrodite sperm are used for self-fertilization until those sperm are depleted, defining the self-fertile period, but hermaphrodites continue to produce oocytes that can be fertilized by male sperm. For most experiments here, worms were grown at 15°C and adults (A1-A5) were synchronized from fourth stage (L4) larvae (arrow in timeline). **B.** Diagram of pachytene germ cells in cross-sectional (left) and surface (right) views. Each germ cell is connected with the gonad core through an opening called a ring channel. Nuclei have large nucleoli (no) that occupy most of the nuclear volume, and the paired homologous chromosomes (Chr, blue) occupy the space between the nucleolus and the envelope. Perinuclear P granules (Pg, white) are associated with clustered nuclear pores (red) and overlie channels between each set of chromosomes. Cer1 GAG particles concentrate on stable microtubules which surround the germ nuclei and extend for long distances into the gonad core. **C.** The image shows a single germ nucleus in surface (left) and cross-sectional (right) optical planes; DNA (blue), P granules (PGL-1; green), and nucleoli (FIB-1; red). Note that the perinuclear P granules are aligned above the channels, corresponding to the positions of clustered nuclear pores. In this and selected images below, the DAPI channel is shown in cyan rather than RGB blue for resolution. **D.**TEM images of three pachytene nuclei; the surrounding cytoplasm is false-colored yellow. Brackets indicate presumptive RNPs in the channels between the compacted chromosomes (Chr). Examples of P granules (Pg) are outlined by dashed lines. Scale bars in microns = (C) 1.0; (D) 0.5.

Cer1 viral particles, called GAG particles, are most abundant in adult hermaphrodites cultured at 15°C [24], the temperature used for all experiments in this study unless indicated otherwise; a timeline of development at 15°C is shown for reference (Fig 1A). Hermaphrodites produce and store "self-sperm" during the fourth and final larval stage (L4), then switch to producing oocytes as adults (A). The self-sperm are largely depleted by adult day 4 (A4); thereafter, oocytes can only be fertilized by sperm from males. Each of the two arms of the hermaphrodite gonad resembles an elongated, U-shaped cylinder lined with about 1000 germ cells (Fig 1A; for general reviews of gonad biology see [38,39]). Germ cells develop in a linear sequence beginning with a mitotic or proliferation zone of germline stem cells at the distal tip of the gonad; germ cells progress through the successive stages of meiosis after leaving the zone. GAG particles first appear in early pachytene, and accumulate in enormous numbers throughout the pachytene region [24]. The gonad is syncytial; each germ cell is connected to a large, shared region of cytoplasm called the gonad core (Fig 1A and 1B). Cytoplasmic materials flow longitudinally through the gonad core toward and into expanding oogonia, which cellularize to become oocytes (Fig 1A) [40]. Pachytene germ nuclei have large nucleoli that occupy most of the nuclear volume; the duplicated and paired homologous chromosomes (tetrads) are distributed in a thin shell of nucleoplasm between the nucleolus and the nuclear envelope (Fig 1B and 1C). In general models for nuclear architecture, the nucleus is thought to resemble a sponge-like body of inactive/silenced chromatin perforated by channels of transcriptionally-active chromatin [41,42]. *C*. *elegans* pachytene nuclei appear to conform with this model, with the compacted, paired chromosomes separated by channels which contain nascent mRNA (Fig 1B and 1C) [43]. By transmission electron microscopy (TEM), the channels often contain small and irregular, electron-dense bodies that likely represent ribonucleoprotein (RNP) granules (Fig 1D, brackets). Most of the mRNA in germ nuclei is exported through clusters of nuclear pores and perinuclear assemblages of proteins, called nuage or P granules, which overlie the nuclear channels (Fig 1B and 1C; reviewed in [44]).

### Cer1 GAG contains a Capsid-like domain

**Fig 2:**
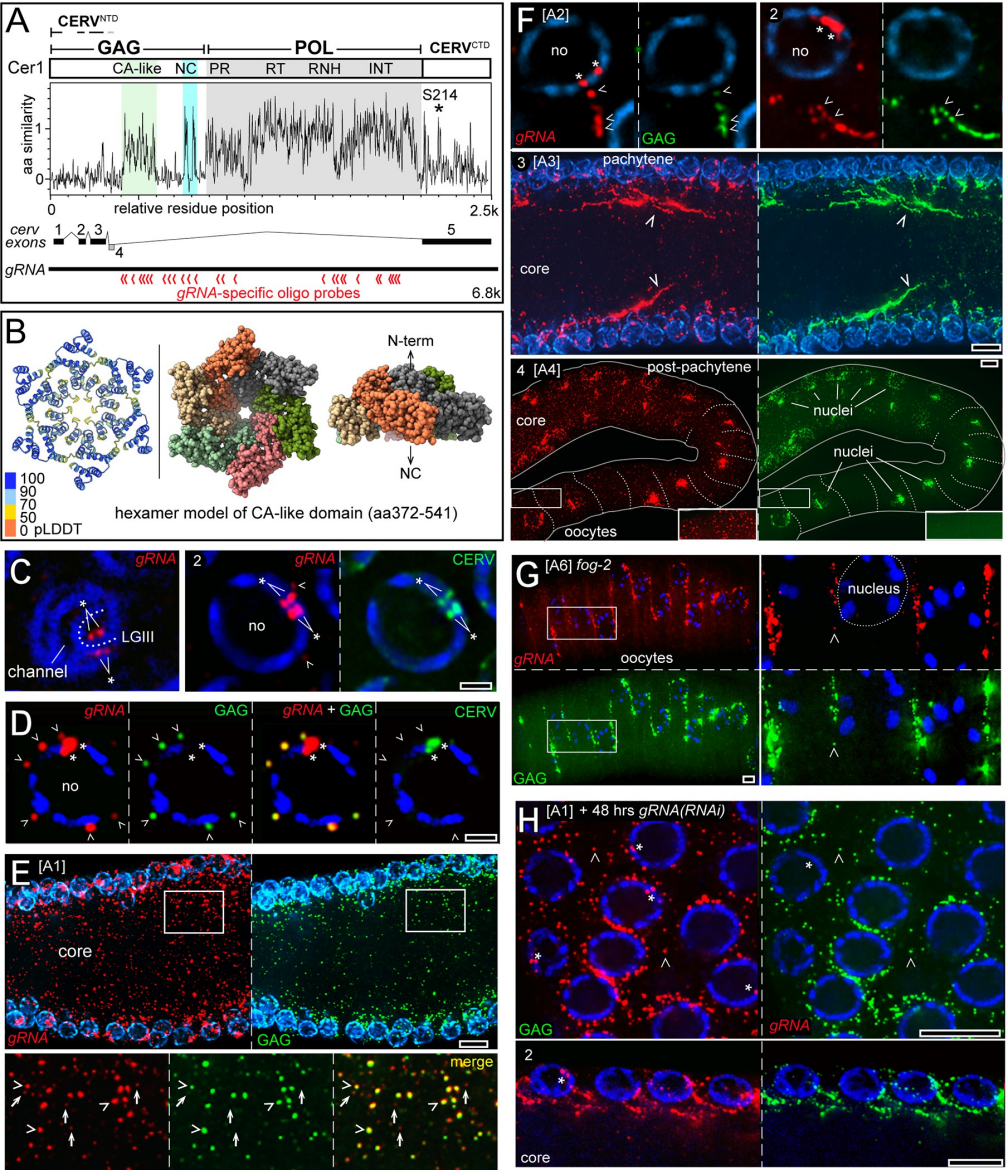
Cer1 GAG and gRNA. **A**. The protein products of Cer1 are diagrammed at top; the GAG polyprotein contains a nucleocapsid domain (NC) and a capsid-like (CA-like) domain defined in this study. The POL protein contains protease (PR), reverse transcriptase (RT), ribonuclease H (RNH) and integrase (INT) domains; boundaries of each domain are described in S1 Fig, legend. The N-terminal half of CERV shares three peptide sequences with GAG, and one unique peptide; splicing joins the four exons encoding these peptides to a fifth exon encoding the C-terminal half of CERV. The plot represents conservation of aligned Cer1 sequences from *C*. *elegans*, *C*. *nigon*, *C*. *remanei*, *C*. *zanzibari*, *C*. *latens*, and *C*. *inopinata*; the alignment for the GAG and CERV regions is shown in S1, S2, and S3 Figs. The plot was generated using EMBOSS Plotcon [130], which computes local similarity from a sliding window, here 4 amino acids; higher values on the y axis indicate higher conservation. The position of the phosphorylated residue S214 described in the text is indicated (asterisk). The red chevrons at bottom indicate the positions of gRNA-specific oligo probes used for smFISH; oligo sequences are provided in S2 Table. **B.** The ribbons/slab model at left shows the AlphaFold prediction for a hexamer of the CA-like domain of Cer1 GAG (amino acids 372–541) color coded as per the AlphaFold pLDDT table, a per-residue estimate of confidence on a scale of 0–100 [131]. The middle and right images show the space-filling model of the same hexamer with arbitrary coloring for each subunit. The model has a dome shape; the middle image shows a view inside the dome, and the image at right shows a side view with arrows indicating the relative positions of the N-terminal and NC regions of GAG. **C.** The left panel shows a near surface plane of a pachytene germ nucleus after smFISH for Cer1 gRNA (red). Four foci of Cer1 gRNA are visible, two on each side of the presumptive LGIII homologs (dotted line). Panel 2 shows a cross-sectional plane through a second germ nucleus; note that the four gRNA foci are coincident with CERV (see below). Arrowheads indicate the relatively low signals from cytoplasmic gRNA. **D.** Image of a single pachytene germ nucleus in an animal raised at 25°C but shifted to the permissive temperature of 15°C for 45 minutes before fixation; the image shows gRNA, GAG, and CERV as indicated. The gRNA exposure was increased relative to Fig 2C to visualize the cytoplasmic foci of gRNA (arrowheads). The increased exposure typically obscures the four foci of nuclear gRNA, such that there appears to be only two, larger foci (asterisks). Most of the perinuclear gRNA and GAG foci overlie nuclear channels, where perinuclear P granules are localized. Note that CERV is localized to the nuclear foci of gRNA, but not the perinuclear, cytoplasmic foci of gRNA. **E.** Low magnification of the pachytene region of an A1 gonad, showing GAG and gRNA dispersed throughout the gonad core; the image is a 3-micron Z-projection. The insets at bottom show that many of the brighter foci of gRNA (arrowheads) colocalize with GAG particles while the dimmer foci (arrows) do not. **F.** Panels 1 and 2 shows linear arrays of cytoplasmic gRNA foci (arrowheads) near pachytene germ nuclei; most of the cytoplasmic but not nuclear (asterisks) foci of gRNA colocalize with GAG particles, which have been shown to form linear arrays through association with microtubules (Fig 1B and [24]). Small arrays are evident beginning at the A2 stage (panels 1 and 2), but additional large, linear aggregates occur in A3 and older animals (panel 3). Panel 4 shows gRNA in the post-pachytene region of the gonad where oogonia increase in size and cellularize as oocytes. gRNA and coincident GAG particles in this region become heavily concentrated around nuclei (see also S1 Video). The oogonia and oocytes also contain dispersed, cytoplasmic gRNA without coincident GAG particles; cytoplasm signals in the boxed regions are shown with increased exposures in the insets. **G.** Arrested oocytes in the proximal arm of an A6 *fog-2(q72)* gonad, shown as a 4-micron Z-projection; the inset shows a higher magnification of the boxed region in three oocytes. Cytoplasmic gRNA and coincident GAG particles are concentrated at the cortical region of each oocyte; note that there is relatively little cytoplasmic gRNA that is not associated with GAG. **H.** Wild-type gonad exposed to *gRNA(RNAi)* for 48 hrs beginning on A1. Panel 1 shows a surface view of pachytene germ nuclei, and panel 2 shows a longitudinal view of the gonad at lower magnification. The cytoplasmic foci of gRNA (arrowheads) are generally coincident with GAG particles (see also S5 Fig). Scale bars in microns = (C, D) 1.0; (E-H) 5.0.

The predicted Cer1 GAG protein is 690 amino acids, which is larger than typical retroviral GAG proteins; for example, GAG proteins from Ty3, HIV-1, and RSV are about 280, 500, and 577 amino acids, respectively [45–47]. Cer1 GAG has a predicted zinc finger nucleocapsid (NC) domain, but no obvious sequence similarity to the matrix (MA) or capsid (CA) domains of retroviral GAG proteins [21]. Cer1 elements are abundant in wild strains of *Caenorhabditis elegans* [20], but are too closely related to suggest functionally important GAG domains. For example, the entire 2272 amino acid polyprotein encoded by Cer1 [QX1794, LGI:3.91] and Cer1 [ECA36, LGI:13.94] differ from the laboratory strain Cer1[N2, LGIII:-0.04] by only 2 and 7 amino acids, respectively. Thus, we used blast searches to identify and align Cer1 elements from five diverse, male-female species of *Caenorhabditis* (S1, S2, and S3 Figs). A summary plot of this alignment (Fig 2A) shows that Cer1 GAG is overall divergent relative to the POL protein, but shows conservation of the NC domain and of a second, previously uncharacterized domain in the position expected for a retroviral CA protein (Fig 2A, green block). We used AlphaFold and ColabFold [48,49] to generate structural predictions for the second domain and used the DALI server [50] to search for related structures in the Protein Database (PDB) [51]. This analysis showed that the predicted structure for the second domain, now designated CA-like, is highly similar to CA structures from diverse retroviruses and endogenous LTR retrotransposons (S4 Fig). Retroviral capsids are assembled from hexamers and pentamers of CA proteins; for example, RSV and HIV capsids contain about 250–300 hexamers of CA and small numbers of pentamers [52,53]. We used AlphaFold to test whether the CA-like domain could multimerize, and found that it was predicted to form hexamers with high confidence scores (Fig 2B). The N-terminal region of GAG preceding the CA-like domain is predicted to form a long alpha-helical coiled-coil (S4 Fig) and does not resemble known structures for retroviral MA proteins. We conclude that Cer1 GAG resembles other retroviral GAG polyproteins in having both CA and NC domains. However, Cer1 GAG lacks the MA domain which can target retroviral particles to the plasma membrane for horizontal transmission [14].

Most Cer1 GAG particles are associated with microtubules, and show complex region- and stage- specific patterns of localization that are proposed to result from a load/release/transfer mechanism [24]: First, newly formed GAG particles in the pachytene region are proposed to load onto a stabilized subset of gonad microtubules; this binding anchors the particles against cytoplasmic flow (Fig 1A) and allows particle accumulation in the core. Second, the accumulated GAG particles are released abruptly as germ cells exit pachytene and microtubules become destabilized. Finally, the released GAG particles transfer to dynamic microtubules and move toward and around oogonia nuclei. We used Green Fluorescent Protein (GFP) to examine the dynamics of particle localization in live animals (S1 Video). GFP particles were present in the expected spatial and temporal patterns in adult hermaphrodite gonads, but were not detected in somatic cells (neurons, muscles, pharyngeal cells, or intestinal cells; n>100 adult hermaphrodites). As predicted by the load/release/transfer model, large aggregates of GAG particles persisted with little movement in the pachytene region, but individual particles moved toward and around nuclei once germ cells exited pachytene (S1 Video).

### Newly exported Cer1 gRNA associates rapidly with GAG

Retroviral genomic RNA (gRNA) can serve as an mRNA template which is translated into GAG and POL proteins, and/or function as the viral genome which is packaged into GAG particles [14]. Previous in situ hybridization experiments on pachytene germ cells showed that Cer1 gRNA is concentrated in two nuclear foci which appear to be at or near sites of Cer1 transcription: The nuclear foci are near the middle of the chromosome, consistent with the Cer1 insertion site in the laboratory N2 strain; there are no nuclear foci in the strain CB4856 which lacks Cer1; and there is only one nuclear focus in heterozygous N2/CB4856 animals [24]. That study showed Cer1 gRNA is highly abundant in gonad cytoplasm, but did not determine whether any of the cytoplasmic gRNA was associated with GAG particles [24]. Because retroviral capsids package only two molecules of single stranded gRNA [54], we re-examined gRNA localization using smFISH (single molecule fluorescence in situ hybridization) in combination with immunostaining for GAG. Mitotic nuclei typically showed 1–2 foci of gRNA, but staining in pachytene nuclei could usually be resolved into four, closely spaced foci which presumably represent each of the four LGIII chromatids (Fig 2C, panels 1 and 2). Numerous, relatively faint foci of gRNA were detected in the cytoplasm, but visualizing the cytoplasmic foci required longer exposures that typically saturated signals from the nuclear foci (Fig 2D, asterisks). There were two classes of cytoplasmic gRNA foci that differed in staining intensity: the brighter foci usually colocalized with GAG (Fig 2D and 2E, arrowheads), but the fainter foci often did not (Fig 2E, arrows). In comparable regions of two A2 gonads, for example, 53% (n = 812) and 73% (n = 1143) of the brighter cytoplasmic foci colocalized with GAG, as did 77% (n = 2103) of the brighter foci in an A4 gonad. To address where newly exported gRNA first associates with GAG, we took advantage of the fact that gRNA export is dependent on temperature: Animals cultured at 25°C have nuclear gRNA, but lack cytoplasmic gRNA and GAG expression [24]. We found that cytoplasmic gRNA and GAG could both be detected within 45 minutes after 25°C adults were downshifted to the permissive temperature of 15°C (Fig 2D). gRNA and coincident GAG were often localized to perinuclear foci (Fig 2D, arrowheads), most of which were in the expected positions for P granules (Fig 1B and 1C). This result suggests that GAG can associate with newly exported gRNA trafficking within or emerging from P granules.

There was a relatively uniform distribution of cytoplasmic gRNA in the gonad core of A1 animals (Fig 2E). By contrast, older adults showed a progressive accumulation of gRNA in lines or giant linear aggregates in the pachytene region, and in perinuclear arrays in the post-pachytene region (Fig 2F). These aggregates colocalized with GAG (Fig 2F), consistent with the expected concentration of Cer1 capsids on microtubules [24]. In addition to perinuclear localization of gRNA and GAG particles, oogonia and oocytes contained substantial amounts of cytoplasmic gRNA which did not appear to localize with GAG (Fig 2F, insets in panel 4). Retroviral capsids protect gRNA from antiviral factors in the host cytoplasm, in addition to their roles in viral assembly and transmission [55]. Thus, we wondered whether cytoplasmic gRNA was protected by an association with GAG. For this analysis we examined gRNA in the oocytes of *fog-2(q72)* mutant gonads: Oocytes are transcriptionally quiescent and cannot synthesize additional gRNA, and because oocytes are cellularized they cannot take up additional gRNA from the gonad core (Fig 1A) [56]. Wild-type oocytes persist for only a few hours before they are fertilized and laid, but *fog-2* mutants can hold large numbers of unfertilized, arrested oocytes for several days [57]. We found that *fog-2* oocytes contained numerous foci of cytoplasmic gRNA through at least A6; nearly all of the gRNA colocalized with GAG particles near the plasma membranes (Fig 2G) where microtubules become concentrated [58]. Because many of the *fog-2* oocytes in A6 adults are expected to have formed on A1, this suggests that GAG-associated gRNA can persist for at least six days. We next asked whether GAG-associated gRNA was resistant to RNAi-mediated degradation. A1 hermaphrodites, which have abundant cytoplasmic gRNA and coincident GAG particles (Fig 2E), were transferred to gRNA-specific RNAi feeding plates for an additional 15, 24, or 48 hours before processing by smFISH and immunostaining. Variable but often large amounts of cytoplasmic gRNA persisted at each timepoint, most of which was coincident with GAG particles (Fig 2H; see S5 Fig for additional data). By contrast, A1 hermaphrodites exposed to RNAi targeting the spliced *cerv* mRNA lost most of the cytoplasmic signal within 6 hours (S5 Fig). Together, these results suggest that Cer1 GAG has structural and functional similarity to retroviral GAG proteins, and appears to package and protect a large fraction of cytoplasmic gRNA.

### CERV is a nuclear protein required for gRNA export and GAG expression

**Fig 3:**
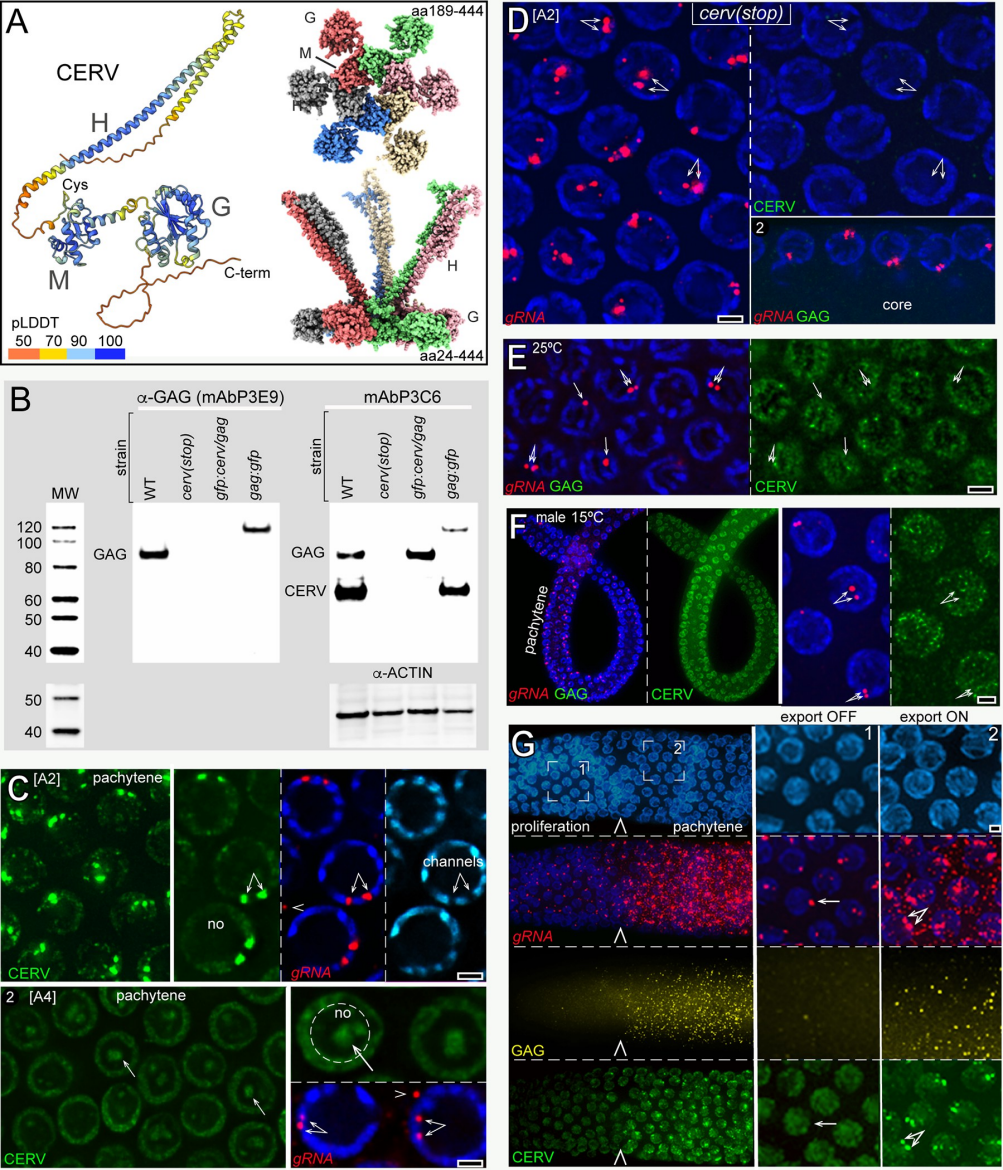
CERV structure and function in gRNA export. **A.** The left image is the AlphaFold-predicted structural model for the complete CERV protein (aa1-517), with the pLDDT color-coded confidence scores as in Fig 2A. The H, M, and G domains and the cysteine-rich loop described in the present study are indicated. Both images at right show different views of a space-filling model of the CERV hexamer; only the indicated amino acids are depicted in the model, and the subunit coloring is arbitrary. The central ring (top right, base view) is built from multimers of the M domain with the conserved Cys loops near the central axis (see also S6 Fig). The G domains extend radially from the M domains by flexible spokes (see also S4 Fig). H domains from adjacent subunits are predicted to bind together in coiled coils that project at variable angles from the ring (bottom right). **B.** Western blot of protein extracts from the indicated strains: *cerv(stop)* = WM746, *gfp*:*cerv/gag* = WM638, and *gag*:*gfp* = WM743 (see S1 Table for details). The blot was probed sequentially with α-GAG, mAbP3C6, and α-ACTIN. Note that the single band recognized by mAbP3C6 in the *gfp*:*cerv/gag* extract is GFP:CERV; this strain does not express GAG:GFP (see analysis in S7 Fig). **C**. Panel 1 (top row) shows A2 pachytene germ nuclei stained for CERV (mAbP3C6); the inset at right shows CERV, gRNA, and DNA (blue and cyan) at higher magnification. Panel 2 shows A4 pachytene germ nuclei stained for CERV. The intense, nuclear foci of CERV have disappeared; CERV is dispersed in the nucleoplasm and present in irregular aggregates at the center of nucleoli (arrows). Note that the level of gRNA in the nuclear foci (double arrows) has decreased and is comparable to the signal from cytoplasmic foci of gRNA (see also S8 Fig). **D.** Germ nuclei from A2 adults with a CERV-specific stop mutation. Panel 1 is an 8 micron Z-projection, showing there is no gRNA detectable in the cytoplasm (compare with the cytoplasmic gRNA visible in Fig 3G, which is a 3 micron Z-projection of similar germ nuclei). The lack of CERV expression is shown in the right half of panel 1. Panel 2 is a 3 micron projection showing the absence of GAG particles in the gonad core. **E.** Pachytene germ nuclei in a wild-type hermaphrodite cultured at the non-permissive temperature of 25°C. The gonad has prominent nuclear, but not cytoplasmic, foci of gRNA, and GAG is not expressed. Note that CERV is present in the nucleoplasm but not concentrated at the nuclear gRNA foci (arrows). **F.** A2 adult male gonad at 15°C. The gonad has nuclear, but not cytoplasmic, foci of gRNA, and GAG is not expressed. Note that CERV is present in the nucleoplasm but not concentrated on the nuclear gRNA (arrows). **G.** A2 adult hermaphrodite gonad at 15°C, showing the boundary (arrowhead) between the proliferation zone (inset 1) where gRNA is not exported, and the pachytene region (inset 2) where gRNA is exported and GAG is expressed. Note that the appearance of gRNA and GAG in the cytoplasm corresponds to where CERV first concentrates on the nuclear foci of gRNA. These images are 3-micron Z-projections, so signals from a few cytoplasmic foci of gRNA are artificially superimposed on the nuclei. Scale bars in microns = (A-G) 1.0.

The Cer1 CERV protein is 517 amino acids and is the product of a spliced, 5 exon mRNA (Fig 2A) [24]. *cerv* exons 1–3 have the same reading frame as *gag*, but *cerv* exon 4 has a unique reading frame (Figs 2A and S1). CERV has no obvious homology to proteins outside of nematodes and, like GAG, is highly divergent between *Caenorhabditis* species (Figs 2A, S1, S2 and S3). CERV contains a potential leucine zipper (Leu-X6-Leu-X6-Leu-X6-Leu) that it shares with GAG, a candidate nuclear localization sequence (KRKK), and a candidate nuclear export sequence MLILADGLRL [59]. CERV does not have an RNA-binding motif recognized by the RPB2GO database [60], but contains a cysteine-rich region that might form a C4-type zinc finger [61]. The AlphaFold model for CERV (P34431; https://alphafold.ebi.ac.uk) predicts three structured domains which are separated by flexible linkers; we term the structured domains H, M, and G (Figs 3A and S4). The H domain is a helix-hairpin-helix which contains the potential leucine zipper, and M and G are both globular domains.

We used AlphaFold and ColabFold [48,49] to generate de novo structural predictions for CERV proteins from each of the five different species of *Caenorhabditis* aligned in S1 Fig and represented by the plot in Fig 2A. Despite considerable sequence variation, the predicted structures were remarkably similar for each of the respective H, M, and G domains (S4 and S6 Figs). We used the DALI server [50] to compare separately each predicted CERV domain with structures in the Protein Data Bank (PDB), and found that the H and M domains did not have a close resemblance to known proteins. By contrast, the predicted structure for the G domain was highly similar to diverse G-proteins and structural mimics of GTPases, but lacked critical residues required for GTP hydrolysis (S4 Fig). We next used AlphaFold and ColabFold to ask whether any of the three CERV domains were predicted to multimerize. M domains from each of the *Caenorhabditis* CERV proteins were predicted to multimerize with high confidence scores: M domains could form closed rings with a minimum of 5 subunits, but larger rings were possible (Figs 3A and S6). Notably, the multimer predictions oriented the potential cysteine finger from each M subunit toward the central axis of the ring (Figs 3A and S6). Multimer models for the nearly complete CERV protein showed the G domains extending from flexible spokes at the periphery of the ring; the H domains from adjacent CERV proteins were linked together at the predicted leucine zippers, and extended at several possible angles from one face of the ring (Fig 3A).

To localize CERV, we began by using Green Fluorescent Protein (GFP) to tag the N-terminus of CERV, which is shared with GAG, and separately tagged the C-terminus of CERV. Unfortunately, subsequent experiments showed that the tagged proteins were only partially functional and often mislocalized in homozygous animals, although the tagged proteins appeared to localize normally in heterozygotes with wild type (S7 Fig and see below). A previous study raised monoclonal antibodies against a partial GAG fusion protein that included one of the peptides sequences shared with CERV (encoded by *cerv* exon 3; Fig 2A) [24]. That study characterized a GAG-specific antibody, mAbP3E9, which recognizes a single band at the expected size for GAG on Western blots of wild-type worm extracts, and recognizes a single, larger band in extracts from a strain expressing GAG::GFP (Fig 3B). We screened the additional monoclonal antibodies on extracts of worm proteins, and found that mAbP3C6 stained GAG plus a second band at 62kD, the approximate size of CERV (Fig 3B). We used mAbP3C6 to immunostain pachytene-stage germ cells, and found that staining was concentrated in two nuclear foci which coincided with gRNA (Figs 2C, 2D and 3C). Longer exposures showed staining throughout the nucleoplasm but, fortuitously, there was little or no detectable staining of GAG particles in the cytoplasm (Fig 2D, arrowheads). This suggests that mAbP3C6 recognizes SDS-denatured GAG and CERV, but cannot recognize GAG in formaldehyde-fixed, non-denatured gonadal tissues. Because *cerv* exon 4 uses a different reading frame than *gag* (Figs 2A and S1) we used CRISPR gene editing to create a *cerv*-specific stop codon in exon 4, *cer1(ne4881stop)*, which would not affect the amino acid sequence of the GAG protein. We found that mAbP3C6 did not stain protein extracts or gonads from the *cerv(stop)* mutant (Fig 3B and 3D, respectively). Thus, mAbP3C6 can be used as a CERV-specific stain on fixed tissues. The *cerv(stop)* gonads had prominent foci of nuclear gRNA, indicating the CERV is not required for gRNA transcription, but gRNA was not detected in the cytoplasm (compare gonad core region in Figs 3D with 2E). Cytoplasmic gRNA in retroviruses is expected to serve as the mRNA template for GAG synthesis; as expected, the *cerv(stop)* mutant lacked GAG expression by Western blot analysis and by immunostaining (Fig 3B and 3D, respectively).

Because the smFISH protocol can recognize single RNA molecules, and does in fact recognize what are likely two gRNA molecules in GAG particles, the complete absence of cytoplasmic gRNA in the *cerv(stop)* mutant suggests that the gRNA is not exported, or exported and degraded instantly. By comparison, germline mRNAs targeted for RNAi-mediated degradation can be detected in perinuclear P granules and in cytoplasm by conventional FISH, and mRNAs targeted for degradation by the nonsense-mediated decay (NMD) pathway can be detected in oocyte cytoplasm [43]. Moreover, multiple unspliced germline mRNAs exit the nucleus and accumulate in the cytoplasm in mutants defective in splicing [62]. These several observations and the finding that CERV is a nuclear protein argue that CERV is required for gRNA export, rather than preventing degradation of cytoplasmic gRNA.

### CERV phosphorylation and localization to nuclear gRNA foci is required for gRNA export

Our experiments and previous studies [24] found multiple conditions where wild-type germ cells have prominent nuclear foci of gRNA, but do not appear to export gRNA to the cytoplasm and do not express GAG: these non-permissive conditions include hermaphrodites cultured at 25°C (Fig 3E), males cultured at any temperature (Fig 3F), and germ cells in the proliferation zone (Fig 3G). We examined these non-permissive conditions and found that in each case CERV was present in the nucleoplasm, but was not concentrated at the nuclear foci of gRNA (Fig 3E–3G). Hermaphrodite gonads have a sharp boundary between the proliferation and meiotic regions where cytoplasmic gRNA and GAG first appear, and this boundary (arrowhead in Fig 3G) coincided with where nucleoplasmic CERV first concentrates on the foci of nuclear gRNA. Thus, the ON/OFF switches in gRNA export and GAG expression are correlated with changes in the association of CERV with nuclear gRNA.

**Fig 4:**
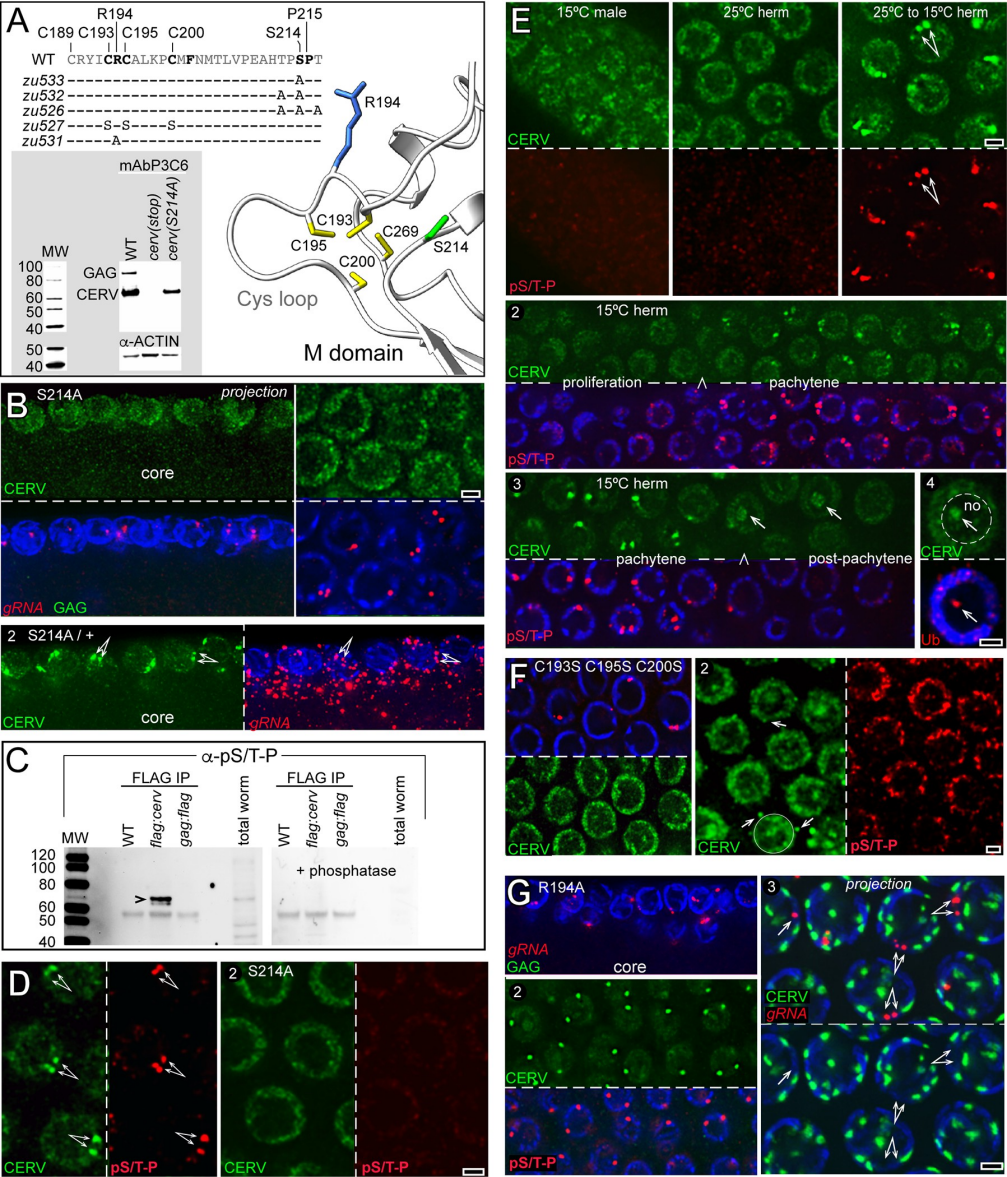
CERV phosphorylation and the cysteine-rich loop are required for gRNA export. **A.**The peptide sequence at top left shows the beginning of the CERV M; residues in bold are invariant in diverse species of *Caenorhabditis* (see also S3 Fig). Most of this region is contained in a flexible cysteine-rich loop (AlphaFold model at right) which faces the central axis of the predicted ring multimers (Fig 3A; see S6 Fig for additional analysis of the M domain). The Cys loop brings multiple Cys residues into close proximity: predicted distances between the cysteine sulfur atoms are C195-C193 (3.3 Å), C193-C200 (3.8 Å), C200-C269 (3.9 Å), and C269-C195 (3.3 Å) (ChimeraX [132]). The blot (inset) shows protein extracts from the following strains: WT, WM746 [*cerv(stop)*], and JJ2706 [(*cerv(S214A)*]; see S1 Table for strain details. The blot was probed with mAbP3C6, which stains a prominent CERV band and a weaker GAG band in the wild-type extract. The *cerv*-specific STOP mutation eliminates both the CERV and GAG band, while the S214A substitution eliminates only the GAG band. **B.** Immunostained gonads from a homozygous mutant with a CERV S214A substitution (panel 1), and from a heterozygous strain with the same mutation plus a wild-type copy (panel 2). **C.** Immunoprecipitation assays of FLAG-tagged CERV and FLAG-tagged GAG followed by Western Blot analysis. Extracts are from WT worms, a strain with a FLAG tag on the N-terminus of CERV (WM894), and a strain with a FLAG tag on the C-terminus of GAG (WM895); see S1 Table for strain details. The extracts were immunoprecipitated with an anti-FLAG antibody and blotted; a duplicate blot is shown at right after treating the same extracts with phosphatase. Both blots were stained with the α-pS/T-P antiserum; the arrowhead points to a band at the predicted size of CERV that is absent after phosphatase treatment. **D.** Germ nuclei in an A2 wild-type gonad (panel 1) and a CERV S214A mutant (panel 2) stained for CERV and phospho-S/T-P. Note that the S214A mutant nuclei fail to stain with α-pS/T-P and CERV is present in the nucleoplasm but not concentrated into foci. **E.** Panel 1 shows different types of germ nuclei as listed after immunostaining for CERV and α-pS/T-P. Note that CERV only concentrates into foci after hermaphrodites are shifted to the export-permissive temperature of 15°C, where CERV becomes phosphorylated. Panel 2 shows the gonad boundary (arrowhead) where CERV first concentrates at the nuclear foci of gRNA, and panel 3 shows the subsequent, post-pachytene boundary where the CERV foci disappear. Some CERV in the post-pachytene region localizes to nucleolar inclusions (arrows) that stain positively for ubiquitin (panel 4, red). **F.** Mutant gonad with serine substitutions at each of the cysteines C193, C195, and C200 in the M domain of CERV (*cer1(zu527)* and strain JJ2700, Fig 4A). Panel 1 shows that nuclear, but not cytoplasmic foci of gRNA are present, and that CERV does not concentrate on the nuclear foci. Panel 2 shows that CERV appears to be phosphorylated at S214. Arrows indicate perinuclear foci of CERV that occur frequently in this strain. **G.** Mutant gonad with an R194A substitution in the M domain of CERV (*cer1*(*zu531)* and strain JJ2704). Panel 1 shows that gRNA is present in nuclear but not cytoplasmic foci, and that GAG is not expressed. Panel 2 shows that the nuclei contain bright foci of phosphorylated CERV. Panel 3 is a 4-micron Z-projection to visualize entire nuclear volumes, and shows that nuclei have more than the two expected CERV foci and that none of the CERV foci are coincident with the nuclear gRNA foci. Scale bars in microns (B,D-G) 1.0 micron.

We wondered whether post-translational modifications such as phosphorylation caused nucleoplasmic CERV to concentrate on nuclear gRNA. CERV contains 48 Ser, Thr, or Tyr residues at possible phosphorylation sites (MusiteDeep and NetPhos-3.1). However, only two of these residues were conserved in all five *Caenorhabditis* Cer1 elements (S3 Fig), including Ser214 in the predicted multimerization domain and near the Cys loop (Fig 4A). We used CRISPR gene editing to create a S214A substitution in CERV and found that Western blots of the mutant extracts had the expected CERV band, but lacked GAG expression (Fig 4A). By immunostaining, the S214A mutant germ cells had abundant nucleoplasmic CERV; indeed, the levels of both nucleoplasmic and cytoplasmic CERV appeared higher than in wildtype (Fig 4B and see Discussion). However, CERV was not concentrated on the nuclear foci of gRNA, and neither gRNA nor GAG were detected in the cytoplasm (Fig 4B). By contrast, control heterozygotes showed the normal concentration of CERV on the nuclear foci of gRNA, and gRNA was abundant in the cytoplasm (Fig 4B, panel 2). We used CRISPR gene editing to create additional alanine substitutions for other Ser and Thr residues flanking S214 (Fig 4A), but each of the double and triple mutants appeared essentially identical to the single S214A mutant. We conclude that S214 is essential for CERV to concentrate on nuclear gRNA, and is required for gRNA export and GAG expression.

We next wanted to determine if CERV was phosphorylated at S214. S214 is followed by an invariant proline residue, P215 (Fig 4A), suggesting that S214 might be phosphorylated by a proline-directed serine/threonine kinase [63]. Multiple proline-directed serine/threonine kinases function in the *C*. *elegans* gonad, including CDK-1/cyclin-dependent kinase, GSK-3/glycogen synthase, and mitogen-activated kinases such as MPK-1 [64,65]. These kinases are expected to have numerous substrates in the gonad, some of which are known for MPK-1 [66]. We generated a worm strain where the N-terminus of CERV was tagged with the FLAG peptide [67], then probed immunoprecipitated extracts from this strain with a commercial antibody that recognizes phosphorylated Ser/Thr-Pro peptides (α-pS/T-P; abcam ab9344). α-pS/T-P stained a prominent band at the expected molecular weight for CERV (Fig 4C; arrowhead). Control experiments (Fig 4C) showed that (1) the band was not detected in immunoprecipitated extracts from wild-type worms which lacked the FLAG tag, (2) the band was not detected in extracts from worms with a FLAG tag on the C-terminus of GAG, and (3) α-pS/T-P did not detect the band when the extracts were pre-treated with phosphatase.

We found that α-pS/T-P showed intense staining of nuclear foci that coincided with the nuclear foci of CERV and gRNA in pachytene germ cells (Fig 4D, panel 1), but showed relatively little staining in the gonad cytoplasm. Because α-pS/T-P is expected to stain several germline-expressed phosphoproteins (see above) and stained multiple bands with comparable intensity in worm extracts (Fig 4C), the prominence of the nuclear foci might result from CERV concentration rather than abundance. α-pS/T-P showed only diffuse, nucleoplasmic staining in S214 mutant hermaphrodites (Fig 4D, panel 2), indicating that the staining of nuclear foci in wildtype is specific for CERV. α-pS/T-P showed only diffuse, nucleoplasmic staining in wild-type males and 25°C hermaphrodites, where CERV does not concentrate on the nuclear foci of gRNA and gRNA is not exported (Fig 4E). By contrast, α-pS/T-P stained the nuclear foci of CERV and gRNA within 1 hour after hermaphrodites raised at 25°C were downshifted to the export-permissive temperature of 15°C (Fig 4E). Similarly, CERV phosphorylation at S214 correlated with the gonad boundary where CERV first concentrates on nuclear gRNA (Fig 4E, panel 2; compare Fig 3G). After germ cells exit pachytene they decrease and eventually cease transcription [56]. The level of phosphorylated CERV diminished abruptly as germ nuclei exited pachytene, coincident with the disappearance of the CERV foci (Fig 4E, panel 3). In the same region, non-phosphorylated CERV accumulated in irregular bodies in the interior of the nucleolus (arrows) which stained positively for ubiquitin (Fig 4E, panel 4). We conclude that S214 phosphorylation is essential for CERV to concentrate on nuclear gRNA, and that this concentration is strongly correlated with gRNA export and GAG expression.

S214 is near the candidate Cys finger in the M domain, which includes invariant cysteine and arginine residues (Fig 4A; see also alignment in S3 Fig). We generated a triple mutant, *cer1(zu527)*, with the cysteines C193, C195, and C200 mutated simultaneously to serine (Fig 4A). CERV was phosphorylated and expressed at abnormally high levels in the triple mutant nuclei, but CERV did not concentrate on the gRNA foci and GAG was not expressed (Fig 4F). The triple mutant also had bright cytoplasmic foci of CERV (Fig 4F, arrows) as observed infrequently in the S214 mutant but not in wildtype. We next generated a mutant, *cer1(zu531)*, with an R194A substitution in CERV (Fig 4A). Like the S214A and triple cysteine mutants, the R194A mutant had nuclear foci of gRNA, but little or no cytoplasmic gRNA or GAG expression (Fig 4G, panel 1). Based on the above results, we anticipated that CERV would not be concentrated into nuclear foci, but instead found that CERV was concentrated in extremely bright foci that stained intensely with α-pS/T-P (Fig 4G, panel 2). However, the mutant nuclei typically had more than the two foci seen in wildtype, and none of the CERV foci colocalized with nuclear gRNA (Fig 4G, panel 3). Thus, these foci possibly represent non-functional aggregates of CERV. Together, our results suggest that gRNA export and GAG expression require S214 phosphorylation and invariant residues in the candidate Cys finger of the M domain.

### CERV transitions from small nuclear foci to giant rods

**Fig 5:**
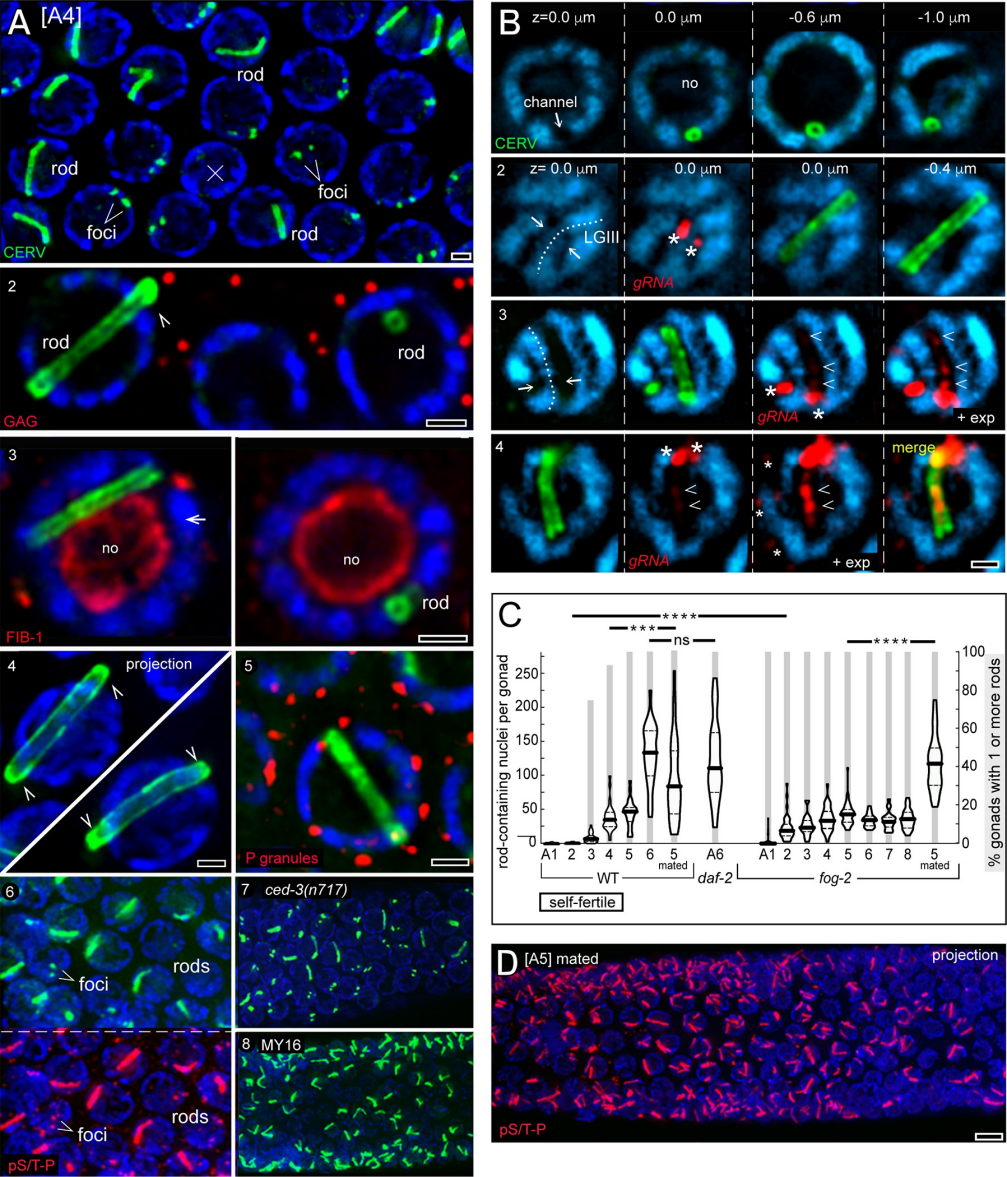
CERV rods in germ nuclei. **A.** Panel 1 is a 3-micron Z-projection of an A4 gonad, showing nuclei with CERV rods in addition to nuclei with CERV foci. Note that the apoptotic cell (x) does not have a CERV rod. Panel 2 compares the sizes of nuclear rods, seen in both longitudinal (left) and cross-sectional (right) profiles, with the sizes of cytoplasmic GAG particles (red). The arrowhead indicates one end of a rod that appears to protrude slightly from the nucleus; additional examples of protruding rods are shown in panel 4. Panel 3 illustrates that rods are associated with the periphery of the nucleolus (FIB-1, red). Panel 5 shows a rod-containing nucleus with perinuclear P granules, which are lost in early apoptosis. Panel 6 shows that the rods are highly phosphorylated, similar to CERV foci. Panel 7 shows CERV rods in an apoptosis-defective *ced-3(n717)* mutant, and panel 8 shows CERV rods in the wild strain MY16 with a Cer1 insertion on LGX [20]. **B.** Panel 1 shows serial optical sections of a single nucleus, demonstrating that the rings of CERV represent cross sections of CERV rods (see also S2 Video). Note that both ends of the CERV rod appear to fill the nuclear channel, but the middle of the rod (at z = -0.6 microns) shifts asymmetrically toward the nucleolus. Panels 2 and 3 indicate the paired LGIII homologs (dotted line) between the two nuclear foci of gRNA (asterisks), and show that the CERV rod localizes to only one of the two flanking nuclear channels (arrows). Increased exposures (+ exp) show gRNA in some rods (panels 3 and 4, arrowheads). **C.** The plot shows the total number of rod-containing germ nuclei per gonad (left vertical axis) and the percentage of gonads with at least one rod-containing nucleus (right vertical axis, grey bars). Mated animals in the wild-type and *fog-2* mutant series were marked and mixed with wild-type males 24 hours before processing; mating was confirmed for each gonad by sperm in the spermatheca, but this experiment did not determine when mating occurred. P-values were calculated using nonparametric Mann-Whitney U test and graphing was performed using GraphPad Prism software version 9.5. **** *P*< 0.0001, ***P ≤ 0.01, ns = not significant. **D.** Gonad of a mated wild-type animal at A5 showing large numbers of CERV rods, all of which stained positively with α-pS/T-P. Scale bars in microns = (A,B) 1.0; (D) 5.0.

By the A4 stage the nuclear foci of CERV diminish or disappear in many pachytene germ nuclei, and some CERV accumulates in nucleolar bodies (Fig 3C, panel 2). The nuclear foci of gRNA persist in A4 germ cells, but often with lower signal intensities that are comparable to cytoplasmic foci of gRNA (Fig 3C, panel 2; see S8 Fig for additional data). In both respects, this class of A4 pachytene nuclei closely resembles younger nuclei exiting pachytene (Fig 4E; panel 3, arrows). Remarkably, however, a second class of A4 pachytene nuclei shows a very different change in CERV localization: The overall level of CERV appears to increase rather than decrease, and CERV forms giant, rod-shaped nuclear structures (Fig 5A, panel 1). The CERV rods have a cylindrical geometry with apparently closed ends; they resemble circles in cross section, and appear as two parallel lines in longitudinal section (Fig 5A, panels 2,3 and S2 Video). CERV is concentrated along the walls of the cylinder, but additional and variable amounts of CERV can occur in the interior. The rods make broad contacts with the periphery of the nucleolus, but never appear to penetrate the nucleolus (Fig 5A, panel 3 and S2 Video). The rods are much larger than CERV foci or cytoplasmic GAG particles (Fig 5A, panel 2): rods are generally 0.4–0.6 microns in diameter (S1 data) and have variable lengths, but usually extend the entire nuclear diameter (4.0 to 5.5 microns). Some rods slightly exceed the nuclear diameter and are either curved (Fig 5A, panel 1) or are associated with small protrusions of the nuclear surface (Fig 5A, arrowheads in panels 2 and 4). The CERV rods stain intensely with α-pS/T-P, similar to the nuclear foci of CERV (Fig 5A, panel 6). Germ cells with CERV rods could be adjacent to, or surrounded by, germ cells which only had CERV foci or diffuse, nucleoplasmic CERV (Fig 5A, panel 1). This heterogeneity was surprising because pachytene germ cells are syncytial and interconnected by ring channels (Fig 1A); they only cellularize during apoptosis, which happens to many germ cells for reasons that are largely unknown [68,69]. However, CERV rods did not appear to result from apoptosis: First, most apoptotic germ nuclei did not contain CERV rods (Fig 5A, "x" in panel 1). Second, nearly all rod-containing nuclei had perinuclear P granules (Fig 5A, panel 5), which normally are lost during the earliest stages of apoptosis [69]. Finally, *ced-3(n717)* mutants which lack germ cell apoptosis had numerous rod-containing germ nuclei (Fig 5A, panel 7). CERV rods were not present in the *cerv(stop)* mutant, or in the mutants with S214A or R194A substitutions in CERV, or in the CB4856 Hawaiian strain of *C*. *elegans* which lacks Cer1 [24]. We examined 7 wild strains of *C*. *elegans* with novel insertions of Cer1 [20] and found that each of these contained variable numbers of germ nuclei with CERV rods (Fig 5A, panel 8; *C*. *elegans* strains MY16, EG4946, JU406, MY23, CB4507, CB4932, and CX11307). Thus, CERV rods appear to be a general feature of the Cer1 retroelement.

Most rod-containing germ nuclei in A4 gonads had only one CERV rod (Fig 5A, panel 1): In a set of 518 rod-containing nuclei, 93.3% had one rod, 5.6% had two rods, and 1.1% had three rods. By contrast, nuclei in older, mated animals frequently contained multiple rods (S8 Fig). The vast majority of rods in A4 gonads aligned with nuclear channels, either filling the channel or displaced toward the nucleolus (Fig 5B, panel 1). Rods were usually found in only one of the two nuclear channels flanking LGIII, where Cer1 is inserted (Fig 5B, asterisks in panels 2,3). However, a small percentage of rods were in nuclear channels that did not align with LGIII, or that were even perpendicular to LGIII (S8 Fig). Several rods appeared to contain gRNA when imaged at increased exposures sufficient to visualize cytoplasmic gRNA foci (Fig 5B, arrowheads in panels 3,4), but the number of rods with gRNA was highly variable between different gonads in the same preparation.

The number of germ nuclei with CERV rods varied considerably between different animals at the same stage, but showed a general increase between A4 and A6 (Fig 5C and S1 Data). This increase raised the possibility that rods were induced by non-specific, age-related cellular stress. *C*. *elegans* lifespan can be doubled by mutations in the insulin receptor DAF-2 (insulin/IGF-1 receptor), and old *daf-2* mutant hermaphrodites retain many features associated with younger, healthier adults [70–72]. However, the numbers of rod-containing nuclei in A6 *daf-2(e1370)* gonads appeared similar to A6 wild-type gonads (Fig 5C and S3 Video). Rods first appear as hermaphrodites approach the end of their self-fertile period and effectively become females which require mating to produce additional eggs. Indeed, we found that A5 adults which were mated on A4 had significantly more CERV rods than unmated A5 adults (Fig 5C and 5D). Ancestral *C*. *elegans* appears to have been a gonochoric species (separate males and females) which evolved into self-fertilizing hermaphrodites by acquiring the sperm-determining gene *fog-2*; *C*. *elegans* mutants homozygous for *fog-2(null)* mutations are healthy females [73,74]. We found that *fog-2(q71null)* females produced rods much earlier than wild-type hermaphrodites: all of the A1 and A2 *fog-2* gonads had at least some rod-containing nuclei, compared with the absence of rods in A1 and A2 wild-type gonads (Fig 5C). The numbers of rod-containing nuclei in the *fog-2* mutants remained relatively constant from A2-A8 (Fig 5C), and the nuclear foci of gRNA and CERV gradually diminished with age (S8 Fig). However, mating restored high levels of both nuclear gRNA and CERV (S8 Fig), and significantly increased the number of rods relative to unmated controls (Fig 5C).

### CERV rods and the nucleolus

**Fig 6:**
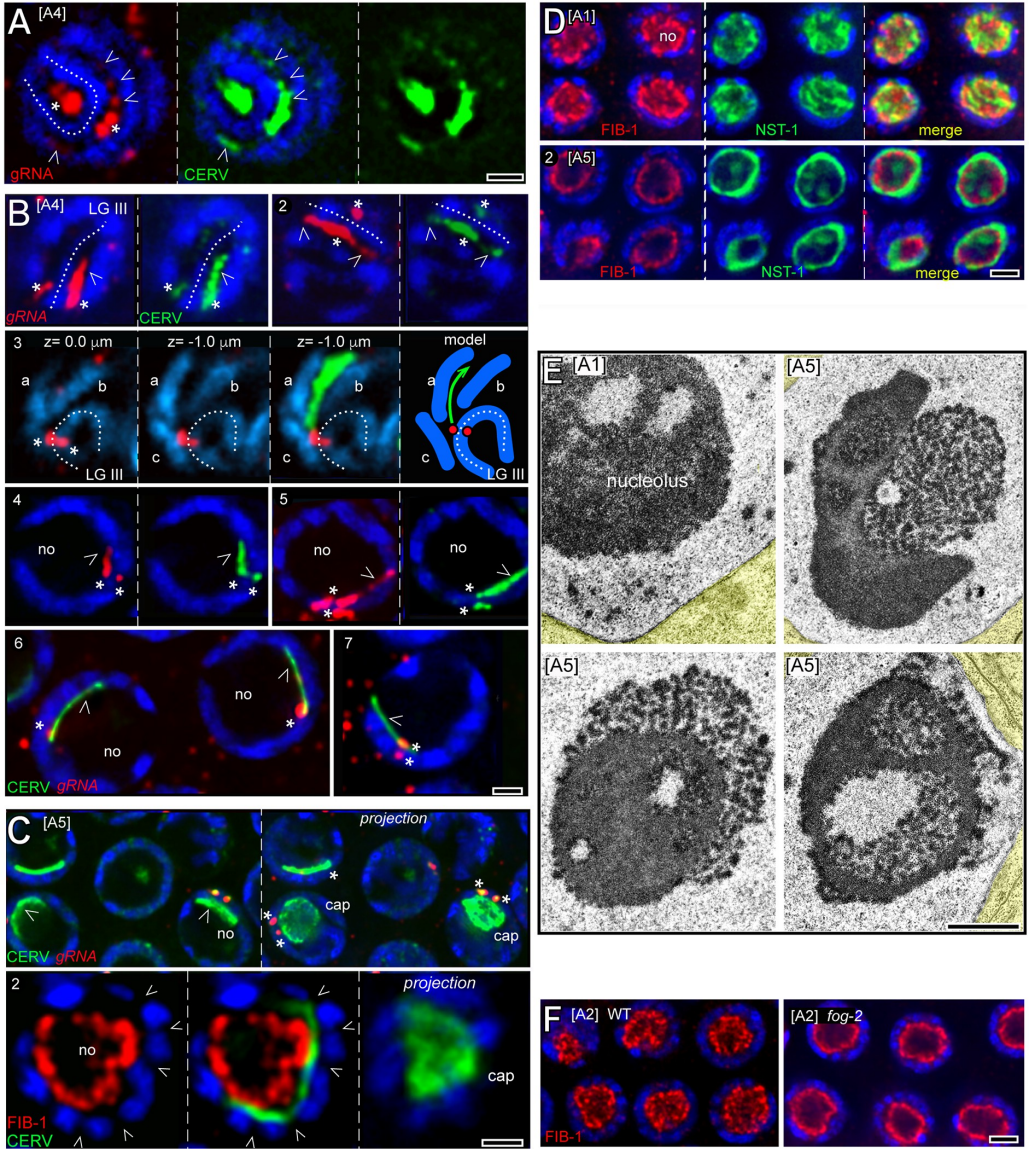
Candidate intermediate stages in the formation of CERV rods. **A.** A4 germ nucleus showing the major nuclear foci of gRNA and CERV (asterisks) with additional signals (arrowheads) in a nuclear channel flanking LGIII (dotted line). **B.** Panels 1 and 2 show near-surface views of germ nuclei with extended, flattened streaks (arrowheads) of gRNA and CERV extending unidirectionally (panel 1) and bidirectionally (panel 2) from the main foci (arrowheads). Panel 3 shows an example of a CERV streak that extends in a channel which is nearly orthogonal to LGIII; a summary reconstruction of the streak and chromosomes is shown at right. Panels 4–7 show multiple examples of flattened streaks of CERV and gRNA at the periphery of the nucleolus. **C**. Panel 1 shows a single focal plane (left) and a 3-micron Z-projection of a group of germ nuclei. The CERV streak at top left remains rectangular in the projected view, but the bottom two streaks (arrowheads) are seen to be much larger oval-shaped "caps" of CERV. Note the main nuclear foci of gRNA and CERV (asterisks) at the perimeters of the caps. Panel 2 shows a germ nucleus with a CERV cap (indicated by the 3-micron Z-projection at right) where the nucleolus is stained for FIB-1/fibrillarin (red). Note that the CERV cap extends below and between multiple nuclear channels (arrowheads), suggesting that the cap is associated primarily with the surface of the nucleolus. **D.** Panel 1 shows dispersed nucleolar components in A1 nuclei, and panel 2 shows segregation of the same components in A5 nucleoli. **E.** TEM images of nucleoli in A1 or A5 wild-type germ nuclei, as listed. Note that the nucleoli in the A5 nuclei have variable patterns of segregation. **F.** Images comparing nucleolar components in A2 wild-type nuclei with the same components in A2 *fog-2* "female" nuclei. Note that the A2 *fog-2* nucleoli resemble older, A5 wild-type nucleoli (see Fig 6D). Scale bars in microns = A-C (1.0), D, F(2.0), E(1.0).

To understand how CERV rods form, we searched for potential intermediate structures. Rings of CERV were frequently visible in germ nuclei, and we considered whether these might template rod formation. However, optical Z-stacks showed that all rings scored were cross-sections of long rods (n = 76; Fig 5B, panel 1; see also S2 Video). The nuclear foci of gRNA did not have a consistent position relative to rod geometry, and could be either at the end or near the middle of a rod (Fig 5B; asterisks in panels 2,3). We noticed that some of the A4 germ nuclei appeared to have slightly elongated foci of CERV and nuclear gRNA (Fig 6A), or highly elongated, flattened streaks of CERV and gRNA (Fig 6B, panels 1 and 2). Similar to CERV rods, the streaks were usually confined to one of the two nuclear channels flanking LGIII, and the streaks could extend either unidirectionally or bidirectionally away from the nuclear foci (Fig 6B; asterisks in panels 1 and 2). The Cer1 insertion in the N2 laboratory strain is near the middle of LGIII, and in some nuclei LGIII made a sharp, U-shaped bend near the nuclear gRNA foci (Fig 6A and 6B, panel 3). In these nuclei, the CERV streak could make a similar, U-shaped bend in one of the curved, flanking channels. In a few cases, however, the CERV streak extended into an adjacent but nearly orthogonal channel (Fig 6B, panel 3); we consider these latter streaks to be possible precursors of CERV rods which do not align with LGIII.

Most of the streaks had widths comparable to the dimensions of nuclear channels. However, the streaks did not fill the channels, and instead were closely associated with the surface of the nucleolus (Fig 6B, panel 4–7). A few streaks were much wider than nuclear channels, and projected views of optical Z-stacks showed that these structures were large and flattened, oval-shaped bodies we term caps (Fig 6C, arrowheads in panel 1). The CERV caps were associated with the surface of the nucleolus, similar to the streaks, but showed no relationship to the boundaries of nuclear channels (Fig 6C; arrowheads in panel 2). This observation suggests that CERV is associating primarily with the nucleolus, rather than channel-specific structures. Interestingly, we found that the structure of the nucleolus changed during the stages when streaks and rods appear: Consistent with results from previous studies [75,76] nucleoli in A1-A2 germ cells had a finely reticulated or net-like appearance, as seen with the nucleolar markers FIB-1/fibrillarin and NST-1/nucleostemin (Fig 6D, panel 1) and as visible by TEM of A1 nuclei (Fig 6E). However, by A5 both FIB-1 and NST-1 appeared to segregate into large, nearly exclusive domains (Fig 6D, panel 2), and TEM images showed a major and highly variable segregation of nucleolar components (Fig 6E). Because CERV rods form earlier in *fog-2* germ cells than in wildtype (Fig 5C), we next examined A2 *fog-2* germ cells and found that they had segregated nucleoli resembling those in older, wild-type germ cells (Fig 6F). Studies in many types of cells have shown that inhibiting ribosomal RNA synthesis causes a segregation or separation of nucleolar components [77], and can cause a relocalization of some nuclear proteins to the periphery of the nucleolus [78]. Thus, streak and rod formation might be initiated by a decrease in rRNA synthesis as adults near the end of their self-fertile period (See Discussion).

### TEM analysis of CERV foci and rods

We wanted to compare the ultrastructure of CERV foci, streaks, and rods using the genetically encoded tag APEX2 (enhanced ascorbate peroxidase 2) [79,80]. With this technique, glutaraldehyde-fixed tissues are treated with 3,3-diaminobenzidine, which is converted to an insoluble polymer near the localized enzyme and visualized by staining with electron-dense osmium. We created a hybrid strain for the analysis which contained three Cer1 elements: Cer1, Cer1(APEX2-CERV), and Cer1(GFP-CERV) (see Methods for rationale and strain construction). The APEX2-CERV hybrid strain was compared to a control strain containing Cer1 plus Cer1(GFP-CERV). For both strains, GFP-CERV expression was used to pre-screen live animals for ones with the largest numbers of rods, and these were pooled for TEM analysis. After processing and embedding, 70 nm thin sections were collected at intermittent intervals throughout the gonads, with additional serial sections from selected regions.

**Fig 7:**
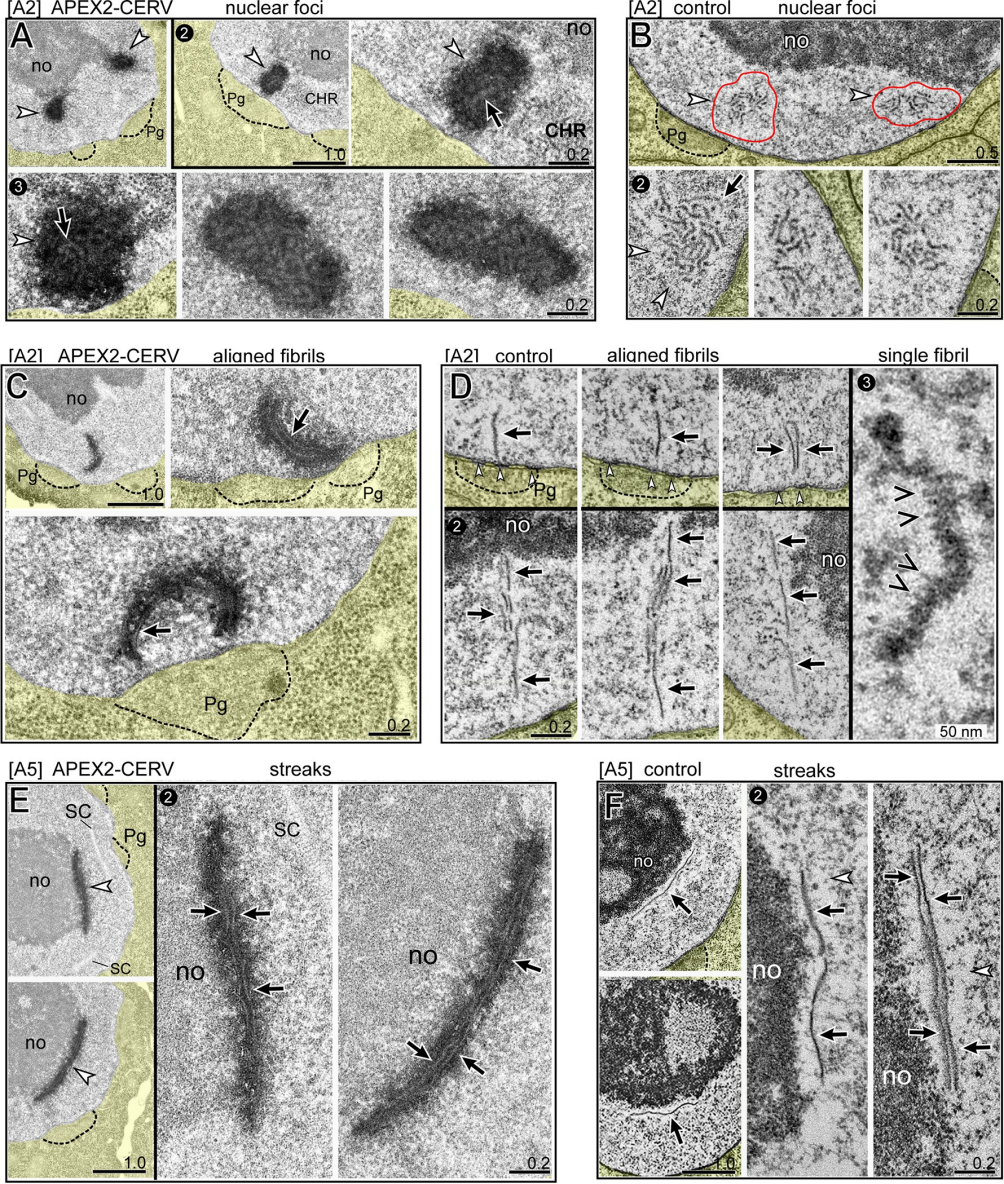
TEM of CERV-containing nuclear structures. **A.** A2 germ nuclei expressing APEX2-CERV. The low magnification images at top show prominent electron-dense regions that appear to be in nuclear channels next to P granules (dotted outlines). Panel 2 shows one such region at high magnification; note the electron-lucent fibrils (black arrows) within the electron-dense region. Panel 3 shows additional examples of the electron-lucent fibrils within APEX2-CERV foci. **B.** A2 control nuclei. Panel 1 shows a nucleus with two clusters of electron-dense fibrils (outlined in red), and panel 2 shows similar regions in other nuclei. White arrowheads indicate electron-lucent regions around the fibrils. **C.** Examples of aligned fibrils in A2 germ nuclei expressing APEX2-CERV; arrows indicate individual, electron-lucent fibrils. **D.** Panel 1 shows three examples of individual fibrils oriented perpendicular to the nuclear envelope; nuclear pores are indicated by small white arrowheads. Panel 2 shows groups of aligned fibrils near the envelope. Panel 3 is a high magnification of a single fibril; note zig-zag appearance (black arrowheads), with striations or flanges that are perpendicular to the long axis of the fibril (see also S9 Fig). **E.** Examples of flattened streaks or caps of APEX2-CERV at the perimeter of nucleoli (no) in A5 germ cells. Note that the streaks contain numerous electron-lucent fibrils (black arrows) which are aligned in a plane parallel to the nucleolar surface. **F.** Control A5 gonads showing electron-dense fibrils (black arrows) surrounded by an electron-lucent matrix (white arrowheads) at the surfaces of nucleoli.

By comparison with our conventional TEM preparations of *C*. *elegans* germ cells [24,43], the APEX2 protocol markedly reduced the background staining of RNA-rich structures such as the nucleolus (compare Figs 7A with 6E). However, many germ nuclei in the APEX2-CERV sample had one or two large and prominent electron-dense foci (Fig 7A, white arrowheads) which were not present in the control sample (Fig 7B). We consider these foci to represent CERV localization: The foci had the expected sizes for CERV foci, they were in nuclear channels, and they were abundant in the A2 sample but largely absent in the A5 sample. At high magnification, the electron-dense foci surrounded a cluster of relatively electron-lucent fibrils (Fig 7A, black arrows). Germ nuclei in the control sample without the APEX2 tag often contained one or two foci with a similar size and location as the APEX2-CERV foci, but which were nearly reciprocal in appearance: these foci consisted of a cluster of electron-dense fibrils surrounded by an electron-lucent zone (Fig 7B, red circles). The clustered fibrils appeared to be randomly oriented and intermingled with small, electron-dense "dots" of the same thickness that likely represent cross sections of fibrils (Fig 7B, panel 2). Because CERV foci are associated with gRNA, we hypothesize that the fibrils represent gRNA molecules. The apparent lengths of individual fibrils varied considerably from an average of about 200 nm up to a maximum of 500 nm (S9 Fig and S1 Data), but these measurements likely underestimate the true average as they were taken from 70 nm thin sections. The fibrils were easily distinguished from presumptive RNP granules which are common in germ nuclei (compare Fig 1D). In addition to the clusters of fibrils, both the APEX2-CERV and control samples contained individual fibrils which were approximately orthogonal to the nuclear envelope and adjacent to nuclear pores and P granules (Fig 7C and 7D, panel 1). Remarkably, some fibrils appeared to be coaligned in narrow, linear tracks near the envelope (Fig 7D, panel 2). At high magnification, individual fibrils typically had a series of fine striations at right angles to the long axis of the fibril, creating a zig-zag appearance that might represent a helical structure (Fig 7D, panel 3; see also S9 Fig).

**Fig 8:**
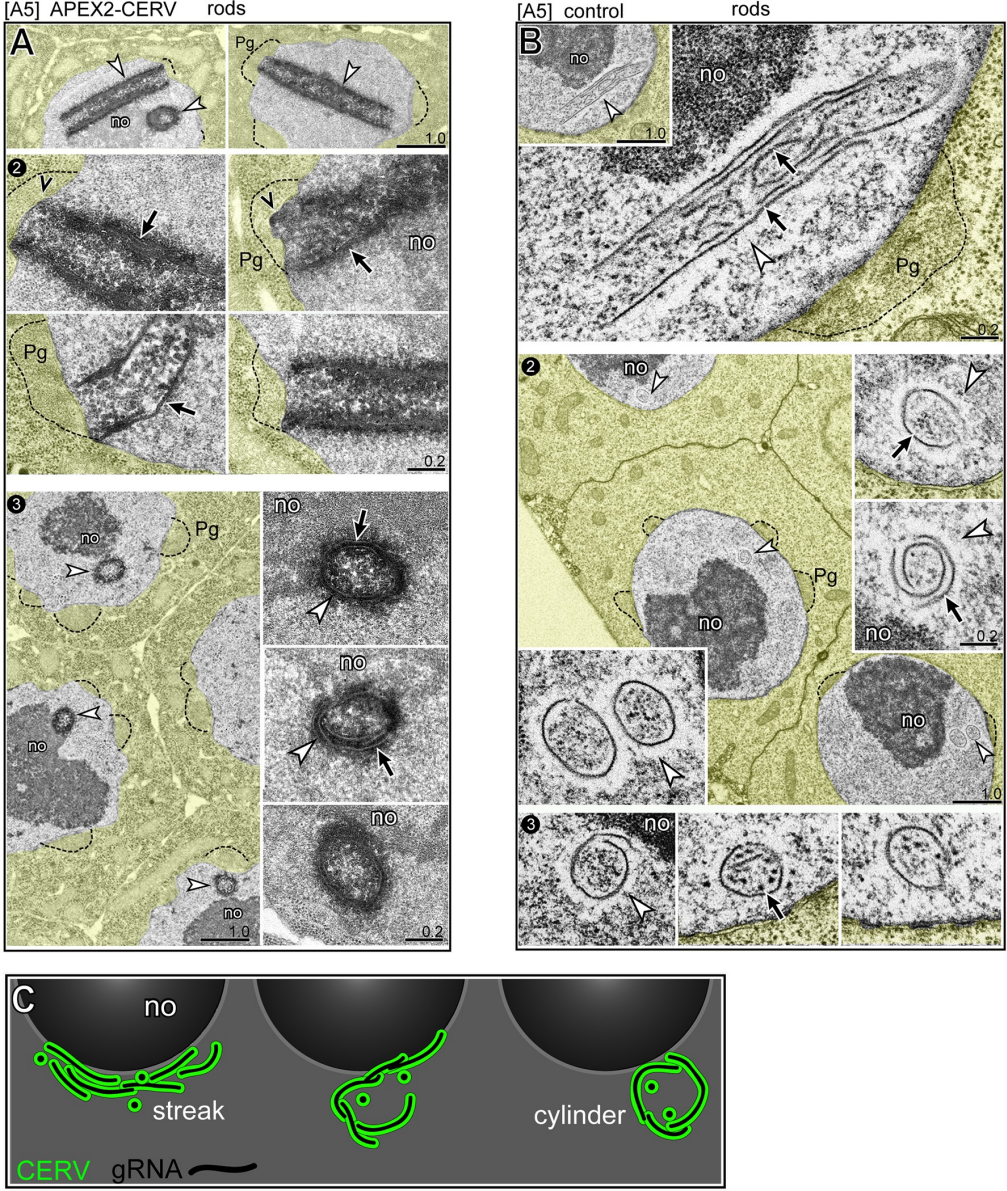
TEM of CERV rods. **A.** Panels 1–3 show longitudinal or cross-sections of APEX2-CERV rods in A5 germ nuclei. Note the electron-lucent fibrils in the rods (black arrows). The top row in panel 2 shows two examples of rods associated with small protrusions (arrowheads) of the nuclear envelope (compare with arrowheads in Fig 5A, panels 2 and 4). In the cross-sectional images (panel 3), note the curvature of the fibrils (black arrows) within the electron-dense matrix (white arrowhead). **B.** Longitudinal or cross-sections of rods in A5 control nuclei; an example of serial sections through a rod is provided in S9 Fig. The rods consist of electron-dense fibrils (black arrows) surrounded by an electron-lucent matrix (white arrowheads). The cross-sectional images of rods in panel 2 are shown as insets at higher magnification; note the curvature of fibrils at the perimeter of the rods. One of the nuclei in panel 2 has a rare double rod which is also seen in immunostained preparations (S8 Fig). Panel 3 shows additional examples of cross-sections through rods. **C.** Speculative model for CERV rod formation, shown in cross-sectional view. CERV and gRNA are proposed to form flattened streaks or caps on the surfaces of nucleoli. Unknown events cause the streaks to roll lengthwise into cylinders, such that gRNA molecules which were previously parallel to the nucleolar surface now become curved. Scale bars in microns as listed.

The APEX2-CERV sample at A5, but not A2, had several examples of electron-dense streaks or caps at the nucleolar periphery (Fig 7E, white arrowheads). At high magnification, the electron-dense streaks contained electron-lucent fibrils which were aligned in the plane of the nucleolar surface (Fig 7E, black arrows). The control sample had streaks or caps of material at the nucleolar periphery with a reciprocal appearance; electron-dense fibrils surrounded by an electron-lucent matrix (Fig 7F, black arrows and white arrowheads, respectively). The A5 APEX2-CERV sample contained several nuclei with longitudinal or cross-sectional profiles of giant, electron-dense cylinders (Fig 8A, panels 1,2 and 1,3, respectively; see S9 Fig for serial section data). As expected for CERV rods, the cylinders were closely associated with the nucleolar periphery, and the ends of the cylinders could be adjacent to slight protrusions of the nuclear envelope (Fig 8A, arrowheads in panel 2; see also Fig 5A, arrowheads in panel 4). The A5 control nuclei had longitudinal and cross-sectional profiles of giant cylinders with similar dimensions, but with a reciprocal appearance; electron-dense fibrils surrounded by an electron-lucent matrix (Fig 8B; black arrows and white arrowheads, respectively). Some fibrils at the periphery, or inside of, a cylinder were oriented parallel to the long axis of the cylinder (Fig 8A, panels 1 and 2 and 8B, panel 1). However, many of the fibrils visible in cross sections of the cylinders had a curvature matching the circumference of the cylinder (black arrows in Fig 8A and 8B). This curvature suggests that rods might form from flattened streaks of CERV and gRNA which rolled lengthwise into cylinders (Fig 8C). This model predicts that intermediate hemicylinders of CERV might be present in some germ cells. Our immunostaining experiments did not detect convincing examples of hemicylinders with the dimensions expected for precursors of typical rods. However, 3/318 A5 germ cells had larger than expected hemicylinders of CERV, and the same population contained a few CERV rods with unusually large diameters (S8 Fig). Thus, we speculate that the large hemicylinders and rods might both be derived from large, ovoidal caps of CERV (Fig 6C, panel 1), while most rods are derived from narrow streaks.

## Discussion

Retroviral gRNA is contained and protected by a protein shell, the capsid, which is assembled from hexamers and pentamers of CA, a subdomain of the GAG polyprotein. Cer1 GAG lacks sequence similarity to CA, but we showed that it contains a CA-like domain with predicted structural similarity to CA and the potential to form hexamers. Thus, Cer1 GAG resembles retroviral GAG proteins in having both CA and NC domains, but lacks sequence or structural similarity with the N-terminal MA (matrix) domain of retroviruses [81]. Most LTR retrotransposons in the Ty3/Gypsy family, which includes Cer1, lack MA proteins and their GAG proteins begin with the CA domain [81]. By contrast, Cer1 GAG has an N-terminal domain which is larger than most retroviral MA proteins (S4 Fig). MA proteins direct the site of capsid assembly; retroviruses such as HIV use MA proteins to assemble capsids at the plasma membrane, but others such as mouse mammary tumor virus (MMTV) assemble capsids in the cytoplasm [82,83]. The N-terminal domain of Cer1 GAG might have a role in targeting to the perinuclear P granule that overlie clustered nuclear pores, where GAG would be positioned to intercept newly exported gRNA. Consistent with this hypothesis, our previous TEM studies showed that Cer1 capsids are often found by P granules in *C*. *elegans*, and presumptive Cer1 capsids in *C*. *japonica* form grape-like clusters on P granules [24]. We showed here that newly exported gRNA appears to associate with GAG at or near P granules in *C*. *elegans* (Fig 2D), but we do not know whether those GAG particles represent entire capsids, or smaller intermediates in capsid assembly. For example, small numbers of retroviral GAG proteins or hexamers are thought to bind gRNA before the GAG-gRNA complex moves to capsid assembly sites [84]. The N-terminal GAG domain of Cer1 might also function in targeting assembled capsids to microtubules, where they accumulate in enormous numbers (Fig 2F). TEM studies have shown that Cer1 capsids are coated with fine, radially-oriented spikes which extend about 12–19 nm [24]; interestingly, the N- terminal GAG domain of Cer1 contains a long, 100 amino acid alpha helix which would be predicted to measure about 15 nm (S4 Fig).

Cer1 GAG particles are highly abundant in adult hermaphrodite gonads, but genetic experiments which detected spontaneous transposition of DNA transposons did not observe novel insertions of Cer1 [85,86]. Endogenous retroelements are expected to accumulate disruptive mutations over time, or become truncated by unequal homologous recombination between LTRs [87]. Thus, the discovery that Cer1 GAG particles have a role in memory transfer strengthens the possibility that Cer1[N2] is a disabled relic, co-opted or repurposed solely for host functions [30]. Our findings that Cer1 GAG particles are not empty, and can instead contain gRNA, supports a view that Cer1[N2] remains intact. Further support comes from recent genomic sequencing of wild strains of *C*. *elegans* which have nearly identical Cer1 elements at different insertion sites ([20] and this study). The failure to detect Cer1 transposition could be because the culture temperature was not optimal for Cer1 expression [24], or that host factors normally restrict transposition [88]. An additional possibility is that Cer1 transposition is disfavored in unmated hermaphrodites: All of the self-progeny of a hermaphrodite will be homozygous for the maternal copy of Cer1 by default, and transposition risks disrupting essential host genes or creating novel anti-sense insertions that silence expression [89]. In natural populations, Cer1 transposition might be stimulated by environmental stresses that challenge host survival [90,91] or by mating with males. Because Cer1 capsids accumulate on stable microtubules, the vast majority of capsids never enter self-progeny [24]. However, our results suggest that the capsids provide a potential reservoir of age- and RNAi-resistant Cer1 genomes that might impact cross-progeny sired by males [20].

### CERV and nuclear export of unspliced gRNA

Intron-containing pre-mRNAs normally are retained in nuclei or degraded in the cytoplasm, but the retroviral life cycle requires some mechanism to export and maintain unspliced gRNA. Moreover, exported but unspliced RNA can trigger gene silencing through poorly understood mechanisms that retroviruses likely overcome [92]. Intron-containing mRNAs often contain premature stop codons, and are degraded in the cytoplasm by the nonsense-mediated decay (NMD) pathway [93]. Most retroviruses have a stop codon between *gag* and *pol*, and require mechanisms to prevent NMD. For example, Rous sarcoma virus gRNA has an RNA stability element which appears to protect against NMD [94]. Cer1 gRNA does not contain a stop codon between *gag* and *pol*, and would not be expected to be targeted by NMD after nuclear export [24]. However, Cer1 gRNA retains conventional *C*. *elegans* splice sequences which are recognized and used to create the spliced *cerv* mRNA [24].

The initial steps in nuclear export begin as pre-mRNAs are loaded co-transcriptionally with proteins in the TREX (Transcription and Export) complex, and are further marked after splicing by exon junction complex (EJC) proteins [95–97]. These complexes have unique core components, and share some subunits such as UAP56 and Aly/REF. Depletion of the *C*. *elegans* homologs of UAP56 or AlyREF allows many unspliced germline RNAs to be exported to the cytoplasm through the CRM1/Exportin pathway, suggesting that these proteins normally have important roles in retaining unspliced RNA in germ nuclei [62]. In principle, therefore, viral gRNAs might become competent for export simply by blocking their association with proteins like UAP56 and Aly/REF. However, retroviruses such as Mason-Pfizer monkey virus achieve export by a secondary structure in the gRNA which directly binds the host nuclear export protein NXF1/NXT1 [98,99]. Other retroviruses such as HIV-1 use an intermediate protein, Rev, to couple the gRNA with the host nuclear export protein CRM1/Exportin; Rev binds and multimerizes on a structured region of the gRNA, and contains a nuclear export sequence recognized by CRM1 [100].

We showed that the Cer1 CERV protein is required for gRNA export: First, a *cerv*-specific STOP mutation or single S214A or R194A substitutions in CERV resulted in an absence of cytoplasmic gRNA and prevented GAG expression. Second, CERV is a nuclear protein which colocalizes with nuclear gRNA (Fig 2C and 2D). Third, our TEM experiments showed that CERV and likely gRNA molecules can localize next to the nuclear envelope by nuclear pores (Fig 7C and 7D), but APEX2-CERV was not detected outside the envelope. Cer1 gRNA export is regulated by ON/OFF switches that depend on sex, developmental stage, cell cycle, and culture temperature; at each switch, export occurs only when CERV colocalizes with the nuclear gRNA. For example, gRNA is not exported at 25°C, but appears in the cytoplasm after a brief shift to 15°C in parallel with CERV concentration on nuclear gRNA (Fig 2D). Similarly, gRNA is exported in meiotic germ cells at the same spatial boundary where CERV first concentrates on nuclear gRNA (Fig 3G). CERV localization to gRNA requires phosphorylation at S214, and we showed that an antibody which recognizes phosphorylated S214 stains CERV at each of the ON switches (Fig 4E). We have not identified the presumptive proline-directed Ser/Thr kinase that phosphorylates CERV, but the finding that phosphorylation does not occur at 25°C raises the possibility that kinase activity is temperature-sensitive; GAG expression has been shown to have a similar temperature-sensitivity in each of several wild strains that express Cer1 [24]. CERV localization to gRNA might have several possible functions that direct or permit gRNA export. For example, CERV has a candidate nuclear export signal, but we have not determined whether CERV functions as a shuttling protein like the HIV-1 Rev protein. Wild-type gonads have very little detectable CERV in the cytoplasm, but CERV might only need to shuttle between the nucleus and perinuclear P granules to achieve export.

Blocking nuclear export can increase the nuclear abundance of some mRNAs. For example, treating *C*. *elegans* with leptomycin B, a specific inhibitor of CRM1/Exportin-mediated nuclear export, increases the level of *tra-2* mRNA in intestinal nuclei [101]. However, none of the several *cerv* mutations described here caused an obvious increase in the level of gRNA in germ nuclei. Conversely, each of the mutations appeared to increase the level of CERV relative to wildtype (Fig 4B and 4F). Thus, one likely possibility is that gRNA splices by default into *cerv* mRNA in these mutants, and that *cerv* mRNA is exported by conventional pathways. CERV is a large protein which lacks sequence or structural similarity to any known retroviral protein, but is predicted to have three structured domains which are conserved in Cer1 elements found in different species of *Caenorhabditis*. The G domain has an interesting resemblance to G-protein structures but is not expected to have similar enzymatic activity (S4 Fig). Thus, the G domain might have originated from a G-protein but been maintained solely to recruit accessory proteins [102]. The M domain has two opposing surfaces with high confidence potentials for binding the complementary surfaces of adjacent M domains (S6 Fig); these interactions could allow CERV to form closed rings with 5 or more subunits, or might create the potential for open, helical stacking (see S9 Fig). Each M domain has a cysteine-rich loop which faces the central axis of the ring and might represent an RNA-binding, C4-type zinc finger (Fig 4A). Structural and biochemical studies will be required to test possible interactions between CERV and gRNA, but we showed here that the invariant cysteines and an arginine in the Cys loop are each essential for CERV concentration at the nuclear foci of gRNA and for gRNA export.

### CERV and nuclear fibrils

Our TEM analysis showed that CERV surrounds electron-dense fibrils in germ nuclei, and we propose that these fibrils represent Cer1 gRNA molecules. Animal nuclei typically contain RNP or mRNP granules such as Cajal bodies, nuclear speckles, paraspeckles, PML bodies, and ribosome intermediates [103–105]. These nuclear granules have diverse roles that include pre-mRNA and ribosomal RNA processing, and presumptive RNP granules are common in *C*. *elegans* germ nuclei (Fig 1D). By contrast, there appear to be very few reports in any system of linear fibrils in nuclei, although viruses often form rods or linear filaments in the cytoplasm [106]. Human cells infected with Rift valley fever phlebovirus develop nuclear fibrils that contain the viral encoded NSs protein, and some amoeba and ciliate nuclei contain electron-dense, helical fibrils of unknown identity that can be up to 700 nm in length [107–110]. The helical fibrils appear to contain protein and RNA, and can be oriented perpendicular to the envelope and adjacent to nuclear pores [107].

The Cer1 gRNA is about 8.4 kb, which is much larger than most mRNAs in *C*. *elegans*; only two germline-expressed mRNAs appear to be larger (encoding HIM-4/hemicentin and HMR-1/cadherin) [111]. The fibrils have some resemblance to electron-dense fibrils which are visible in TEM images of Cer1 capsids [24], and our present study showed that many of the capsids contain gRNA. The nuclear fibrils were often found in prominent clusters in A2 gonads (Fig 7A and 7B); these clusters likely correspond to the nuclear foci of gRNA seen by smFISH (Fig 2C), and thus are at or near the sites of Cer1 transcription. However, individual fibrils could be perpendicular to the nuclear envelope and directly adjacent to nuclear pores, suggesting intermediate stages of nuclear export (Fig 7C and 7D). Fibrils were up to 500 nm in length (S9 Fig), raising the question of what length is expected for an 8.4 kb RNA? Previous studies used electron microscopy to measure contour lengths of single, denatured RNA molecules of various known sizes that were spread on protein films [112]. Those studies showed an average metric of 1 micron per 4.3 kb, implying that a fully unstructured Cer1 gRNA could extend nearly 2 microns. However, most RNAs are thought to be folded; purified RNAs in solution fold into highly compacted structures, and RNAs as large as 5 kb can have 5’ to 3’ end separations of less than 10 nm [113,114]. We found that programs that predict RNA secondary structures, such as RNAfold [115], generate highly folded models for Cer1 gRNA, suggesting that gRNA would not form linear fibrils unless it was associated with factors that restrict folding. For example, the 6.4 kb genomic ssRNA of tobacco mosaic virus is twisted into a helix by multimers of coat protein to create 300 nm, rod-shaped viral particles [116].

A surprising feature of the *C*. *elegans* fibrils is their ability to form linear arrays (Fig 7D, panel 2). FISH experiments in other systems described curvilinear tracks of viral RNA that appeared to extend from sites of transcription toward the nuclear envelope [117,118]. However, the prevailing view is that mRNPs do not travel in linear tracks to nuclear pores, but instead move by random diffusion within channels bordered by compacted chromatin (channeled diffusion) [119,120]. Restricted movements are most apparent with large RNAs such as the 14kb Duchenne muscular dystrophy mRNA [121], and where there are dense chromatin barriers such as *Drosophila* polytene chromosomes [122]. Typical channel widths in *C*. *elegans* pachytene nuclei appear to be about 0.5–0.75 microns, as measured between the DAPI-stained pairs of chromosomes or between the electron-dense chromatin visualized by TEM (Fig 1C and 1D). By contrast, the linear arrays of fibrils have a combined width of less than 0.2 microns, and do not appear to be directly adjacent to compacted chromatin (Fig 7D). These observations raise the possibility that the fibril arrays form by association with unidentified linear structures, such as nuclear actin filaments. We suggest that the large size of the fibrils in *C*. *elegans*, the ability to rapidly induce gRNA export by shifting temperature, and the density of germ nuclei make the gonad an attractive system for analyzing gRNA export in vivo.

### CERV rods and the nucleolus

Several types of retroviral GAG proteins are capable of self-assembly in vitro to form spherical or rod-shaped capsids [93]; retroviral capsids average 100 to 200 nm in size, and the largest known viruses are about 1.5 microns [123,124]. By contrast, CERV rods can be nearly 5 microns in length, and our results suggest that rod morphogenesis involves host structures in addition to any contribution from self-assembly. By TEM, the fibrils in the streaks or caps of CERV are uniformly aligned parallel to the plane of the nucleolar surface (Fig 7E and 7F), but the fibrils visible in cross sections of rods have a curvature matching the circumference of the cylinder (Fig 8A and 8B). As a working model, we propose that rod formation begins as CERV and gRNA localize to the surface of the nucleolus, and that the flattened streaks roll up longitudinally into cylinders (Fig 8C). Additional growth at both ends of the cylinder could extend rod length to match or exceed the nuclear diameter. The CERV streaks and rods that form at A4 have relatively uniform widths, and usually track a nuclear channel flanking the Cer1 insertion on LGIII. However, the caps and many of the streaks of CERV that form in later germ nuclei are not delimited by nuclear channels (Fig 6C, panel 2); at those stages some nuclei have multiple rods, and some of the rods can have atypically large diameters (S8 Fig). A rolling mechanism could accommodate this variation, and explain the rare examples of giant hemicylinders of CERV (S8 Fig).

What causes CERV and gRNA to relocalize from nuclear foci into nucleolar-associated streaks? Previous studies on induced gene expression in *C*. *elegans* germ cells [43] and analogous observations in this report (Fig 2D) suggest that germ cell RNAs in early adults move freely within the network of interconnected nuclear channels. Localized streaks of gRNA and CERV appear on the surfaces of nucleoli at a transitional period in the reproductive cycle as older hermaphrodites deplete their stores of self-sperm and effectively become female. *fog-2* females can produce rods much earlier than wild-type hermaphrodites, suggesting that the female state is relevant to understanding how rods form. We showed that the structure of the nucleolus changes markedly near the time that hermaphrodites become depleted of sperm, and that similar changes occur in much younger *fog-2* females. In particular, the nucleolus loses its reticulated appearance, and the various nucleolar components segregate into largely separate domains. Nucleolar morphology is closely linked with activity, and nucleolar segregation occurs frequently in plant and animal cells that repress rRNA transcription during normal development, or after Pol I transcription is inhibited experimentally by low concentrations of actinomycin D or oxaliplatin [77,125]. Moreover, conditions that induce nucleolar segregation cause a subset of nucleoplasmic proteins to translocate to the nucleolus, often in structures called nucleolar caps [78]. For example, p80 coilin, a protein normally found in Cajal bodies, and the splicing factor PSF both translocate to the periphery of the nucleolus [78].

Sperm-depleted hermaphrodites must wait indeterminant periods before mating, and unfertilized oocytes arrest development until they are activated by signals from male-provided sperm [126]. Relative to young wild-type adults, gonads in sperm-depleted hermaphrodites and unmated *fog-2* females have a reduced mitotic index [127,128] but grow appreciably in size (S8 Fig). This indicates that gonad development is not fully arrested in the absence of self-fertilization. We are not aware of studies that directly examined rRNA transcription in those types of gonads. However, transcriptome studies showed that sperm-depleted hermaphrodites and *fog-2* females have similar gene expression profiles, and that these profiles differ from self-fertile hermaphrodites primarily by the downregulation of ribosomal components [129]. Thus, a decrease in rRNA synthesis could be a molecular signature Cer1 uses to distinguish the self-fertile phase of reproduction from the cross-fertile phase, and serve to trigger an association of CERV and gRNA with the nucleolar periphery (Fig 6B).

### CERV rods; a mysterious structure with unknown function

CERV rods appear to be a widespread, if not ubiquitous feature of Cer1 in wild strains, but we do not understand what function, if any, they serve. The large sizes of the rods raise the possibility that they contain significant quantities of unidentified host proteins or RNAs. Our FISH experiments suggest that only some of the rods contain Cer1 gRNA, but our TEM analysis showed fibrils in essentially all rods. This apparent difference needs to be resolved, but an association with rod components might restrict the penetration of FISH probes, and normally serve to protect the gRNA under harsh environmental or stress conditions. With standard culture conditions, CERV rods only rarely occur in oogonia that will be fertilized by hermaphrodite self-sperm, but rods can be present in many oogonia that have the potential to be fertilized by male sperm (cross-progeny). In natural populations, the optimal adaptive strategy a resident Cer1 uses for the identical self-progeny of a hermaphrodite might be very different than for cross-progeny sired by males. For example, mating could introduce male-derived chromosomes that lack Cer1, and present opportunities for colonization, or that have anti-sense insertions in germline-expressed genes which could silence the host element; the available genome sequences of wild strains of *C*. *elegans* indicate that both types of males are expected in nematode populations [20]. *C*. *elegans* genetics and site-directed gene editing provide powerful tools to dissect rod formation and function, and the expanding database of diverse Cer1 elements provides an important resource for analyzing potential roles in Cer1 ecology. Finally, many LTR retrotransposons have 3’ ORFs of unknown function in the same position as the Cer1 CERV protein, and it will be interesting to learn whether those ORFS have analogous roles in gRNA export and/or contribute to novel viral structures [18].

## Methods

### Worm culture

Nematode were maintained as described [113] with 15°C as the standard culture temperature unless stated otherwise. Plate media was made using Bactopeptone (Difco) and Bacto-agar (Difco) as described [113]. Culture plates were allowed to dry at room temperature for 2 days in the dark, then seeded with a fresh, overnight culture of *E*. *coli* strain OP50. Seeded plates were stored in the dark at room temperature for no more than 5 days. All worm cultures were grown for a minimum of two generations without starving before analysis. All strains used for this study are listed in S1 Table. A minimum of 30–40 animals were analyzed for each experiment reported.

### Antibodies

The following antibodies/antisera were used in this study. Actin (Cell Signaling Technology); Cer1GAG (mAbP3E9) [24]; fibrillarin (abcam ab4566), FLAG (Sigma Aldrich); GFP (Wako); PGL-1 (gift from Susan Strome), GFP (Wako); phospho Ser/Thr-Pro (abcam ab9344); ubiquitin (FK2, Enzo Life Sciences). The secondary HRP-conjugated anti-mouse antibodies were from Life Technologies.

### Analysis of Cer1 elements from wild strains and hybrid strain construction

Previous genomic sequence studies on wild strains of *C*. *elegans* identified a candidate Cer1 LTR at nucleotide 10632248 on LGX in strain ED3046 [20], and we showed this sequence was part of an intact Cer1 element. Cer1[ED3046, LGX] has 491bp 5’ and 3’ LTRs that are identical and differ from the 492 bp LTRs of Cer1[N2, LGIII] only by the absence of an adenosine at position 1, and by a g347t substitution. The remaining differences from Cer1[N2, LGIII] are a957g (5’UTR), t1175a (5’UTR), g5480t (Ribonuclease H domain, Trp to Leu), and t8262ttt (3’UTR). During this analysis, we discovered that ED3046 has a second, unannotated Cer1 element on LGIII, Cer1[ED3046, LGIII]. The second element is nearly identical to Cer1[ED3046, LGX] except for an a7914c substitution that does not change the protein sequence. ED3046 was outcrossed into the Hawaiian wild strain CB4856 which lacks Cer1; expression was monitored by immunostaining for GAG. CRISPR gene editing was used to tag the N-terminus of CERV, and Cer1[ED3046, LGIII; GFP-CERV] was separated from Cer1[ED3046, LGX] by outcrossing 10X against N2 to create strain JJ2669. A strain containing both tagged and wild-type copies of CERV on LGIII was created by crossing JJ2669 into *glo-2(zu455)*; Cer1[N2, LGIII]*unc-32(e189)* hermaphrodites and scoring the homozygous Unc F2 progeny for GFP expression. This strain was backcrossed an additional ten times using N2 males to create the strain JJ2698 used in this study.

### Immunostaining and smFISH

Standard procedures for worm dissection, fixation, and mounting were as described [69,152]. Most images were acquired with a DeltaVision microscope and processed using deconvolution software (GE Healthcare). Images were exported to Adobe Photoshop for contrast/brightness adjustments and cropping. Other images were collected with a spinning disk confocal microscope [Hamamatsu C9100 camera, Nikon TE2000 inverted microscope, Yokogawa CSU-10 spinning disk] using image acquisition software (Volocity 5.3.3, Improvision). Orthogonal projections of optical Z-stacks were generated and analyzed either with Volocity software or with ImageJ.

Cy5- and Cy3-labeled oligo probes for Cer1 *gRNA* and *gfp* are listed in S2 Table (Integrated DNA Technologies). For smFISH plus immunostaining, worms were collected in 30 ul of M9 buffer on taped slides [152] then rinsed in M9 buffer as needed to remove residual bacteria. The M9 buffer was removed and replaced with 30 μl of gonad buffer [48% Leibovitz L-15 (GIBCO), 9.7% Fetal Calf Serum (GIBCO), 1% sucrose, 2 mM MgCl2; adjustments to the osmolality were necessary with different stocks of Fetal Calf Serum, and determined by examining live, dissected gonads under a compound microscope for abnormal shrinkage or swelling. Worms in general were dissected with a single cut below the pharynx to release the gonad. In experiments comparing staining intensities in two groups of worms, one group was dissected near pharynx and mixed with a second group which was dissected near the tail. After dissection, an equal volume was added to the drop of 2X fix [5% formaldehyde (Sigma), 25 mM HEPES (7.4), 40 mM NaCl, 5 mM KCL, 2 mM MgCl2]. The tissues were fixed for 15 mins, then rinsed with RNase-free 0.2M Tris (7.5) for two changes of 1 min then 10 min. The rinse buffer was exchanged with 0.3% Triton X100 in RNase-free PBS (Fisher) and incubated for 15 mins; during this period the worms were further dissected to isolate the gonads and discard other body parts. The detergent solution was removed and replaced with RNase-free PBS for three change, 2 mins each. About 75% of the PBS rinse was removed and replaced with hybridization wash buffer [10% deionized, nuclease-free formamide (Fisher), 2X RNAse-free SSC (Saline-Sodium Citrate, Fisher)] for 2 mins; this solution was removed and replaced with hybridization wash buffer for 10 mins at room temperature. The hybridization wash buffer was removed and replaced with probe mix containing 10% formamide and 90% Stellaris RNA FISH Hybridization Buffer (Stellaris, SMF-HB1) for 4 hours at 37°C in a sealed, humidified chamber. At the completion of hybridization, the gonads were rinsed with several changes of RNAase-free PBS over a total of 10 minutes at room temperature. The PBS was removed and replaced with 50 mM DTT (Dithiothreitol, Sigma) in RNAase-free PBS, then incubated for 30 mins at 37°C in a sealed, humidified chamber. The DTT solution was removed, and the gonads were washed with three brief changes of PBS and incubated in PBS for 10 mins at room temperature. All subsequent steps were as for standard immunofluorescence (above), expect that RNAse-free PBS was used for all washes and to dilute antibodies, and all incubation solutions included 20 units of RNasin Plus (Promega) per 50ul of total solution. After immunostaining, the sample was rinsed in PBS and stained with 1μg/mL DAPI in ddH_2_O for 10 min, then mounted in Vectashield mounting media (Vector laboratories) and covered with a No 1.5 coverslip (VWR).

### TEM and APEX2 staining

N-terminal tags on CERV impair localization and function in homozygous animals, but appear to be localized normally in heterozygous strains with untagged CERV (see S7 Fig). Hence, for the APEX2 analysis we tagged the N-terminus of CERV in Cer1[N2, LGIII], and crossed this strain with JJ2698 described above; GFP-CERV was included in the hybrid for pre-selecting animals with the largest numbers of CERV rods. The APEX2-CERV strain was compared with a control heterozygous strain, Cer1[N2, LGIII]/Cer1[N2, LGIII; GFP-CERV]. Worms were dissected as above in gonad buffer [48% Leibovitz L- 15 (GIBCO), 9.7% Fetal Calf Serum (GIBCO), 1% sucrose, 2 mM MgCl_2_]. After dissection an equal volume was added of freshly prepared fixative [2.0% glutaraldehyde (Electron Microscopy Sciences, EMS), 50mM cacodylate (pH 7.4), 2mM CaCL_2_) for 2 min, then replaced with ice-cold fixative for 1 hour. Processing for APEX2 staining was essentially as described [80] with minor modifications. The fixative was removed, and the sample rinsed with three quick changes of ice-cold 50 mM Cacodylate (7.4), 2mm CaCl_2_, then incubated in ice-cold 50 mM cacodylate (7.4), 2 mM CaCl_2_, 20 mM Glycine for 5 mins on ice. The sample was rinsed four times for 2, 2, 2, and 10 mins in ice-cold 50 mM Cacodylate (7.4), 2 mM CaCl_2_. The rinse solution was removed and replaced with an ice-cold fresh solution of DAB/H_2_O_2_ [500 ul of a frozen/thawed 10X DAB stock (prepared in advance by dissolving 50 mg diaminobenzidine in 10 ml 0.1 N HCL); 1.7 ml 150 mM cacodylate (7.4), 6mM CaCl_2_; 2.8 ml H20; and 5 ul of 30% (wt/wt) hydrogen peroxide]. After a 6–9 min incubation (sufficient to visualize faint, nuclear spots by light microscopy) the DAB/H_2_O_2_ solution was removed and the sample was rinsed three times for 1, 1, and 10 mins with ice-cold 50 mM cacodylate (7.4). The sample was then postfixed/stained with ice-cold 1% osmium (Electron Microscopy Sciences) in 50mM cacodylate (pH 7.4) for 30 mins, then rinsed several times in ddH2O at room temperature. The gonads were then collected, encased in agar and embedded as described [152]. The same procedures but without DAB/H_2_O_2_ were used for the control sample without the APEX2 tag.

### CRISPR/Cas9 genome editing and transgene construction

The Cas9 ribonucleoprotein (RNP) CRISPR strategy [153] was used for genome editing; guide and donor RNAs are listed in S3 Table. Plasmid pRF4 containing *rol-6 (su1006)* was used as co-injection marker. For short insertions like FLAG and deletion mutations, synthesized single strand DNAs were used as the donor; for long insertions like GFP and APEX2 the PCR products were used instead.

### Immunoprecipitation

About 500,000 synchronized adult worms of indicated strains were homogenized by FastPrep-24 (MP Biomedicals) in lysis buffer [50mM Tris-HCl(7.5), 150 mM NaCl, 1% TRITON X-100, 1 mM EDTA, supplemented with 1 mM PMSF and 1 tablet of cOmplete Protease inhibitor (Roche) per 50 ml]. About 500 μg of cleared worm lysates were incubated with FLAG magnetic beads (Pierce) at 15°C for 1 hour. Beads were washed three times with TBST and treated without or with Lambda Protein Phosphatase (New England Biolabs) according to the manufacturer’s instruction.

### Western blot

Worms were lysed with NuPAGE LDS Sample Buffer (Thermo Fisher Scientific), and each lysate sample was loaded into a polyacrylamide gel and separated by electrophoresis followed by transfer onto PVDF membranes (Millipore). Membranes were blocked for 20 min at room temperature with StartingBlock Blocking Buffer (Thermo Fisher Scientific) then incubated with primary antibodies overnight at 4°C. Blots were developed using a Luminata Crescendo Western HRP substrate (Millipore).

## Supporting information

S1 Table List of strains used in this paper. (DOCX)

S2 Table Oligo probes used for smFISH hybridization. (DOCX)

S3 Table Oligo sequences used for CRISPR gene constructions. (DOCX)

S1 Data Individual Tables show measurements for CERV rod diameters (Fig 5), CERV rods per gonad (Fig 5C), and fibril lengths measured by TEM (S9 Fig). (XLSX)

S1 Video Movie of live, dissected gonad showing Cer1 Protease:GFP (strain JJ2506; GFP inserted after Q1031), which is incorporated into GAG particles. The movie represents an elapsed time of 10 mins and shows the transition as germ cells move between the between the mid-pachytene and post-pachytene regions. Most GAG particles in the mid-pachytene region are in large aggregates that show relatively little movement when imaged for as long as 40 minutes. By contrast, GAG particles in the post-pachytene region are highly mobile, with streams of particles moving toward and around nuclei (inset).
(MOV)

S2 Video The video shows successive 0.5-micron optical Z-planes through the pachytene region of an A5 wild-type gonad. The gonad is stained for CERV (green) and FIB-1/fibrillarin (red). Note that FIB-1 is localized near the periphery of most nucleoli, by contrast with the appearance of nucleoli in younger gonads (compare Fig 6D).
(MOV)

S3 Video The video shows successive 1.0-micron optical Z-planes through the pachytene region of a A6 *daf-2(e1370)* gonad; CERV is green and DAPI-stained DNA is white. The first image is a Z-projection of the entire stack.
(MOV)

S1 Fig Alignment of Cer1 polyproteins in *Caenorhabditis* species; amino acids 1–445. The diagram at top summarizes protein domains in Cer1 as described previously [21,24] and extended here (see below), plus an additional capsid-like (CA-like) domain in GAG. M and G subdomains of CERV are indicated and described in the text. The sizes shown for the various POL domains were updated as per InterPro protein family classifications (http://www.ebi.ac.uk/interpro/) [133]: protease (PR, aa735-859, IPR021109), reverse transcriptase (RT, aa1052-1231, IPR000477 plus 1241–1326, IPR043128), and ribonuclease H (RH, aa1327-1429, IPR041373). A previously described integrase (IN) domain in Cer1 (approximately aa1546-1603, IPR041588 plus aa1616-1774, IPR001584) was extended here to aa1873 based on structural prediction: AlphaFold and ColabFold [48,49] were used to generate a structural model for the extended region, which was compared with structures in the Protein Database (PDB) using the Dali server [51]. This analysis showed that the extension had structural similarity to integrase proteins from retroviruses such as Foamy Virus (PDB ID: 5frn-A; Dali Z score 6.8) and Rous Sarcoma Virus (PDB ID:1c0m-B; Dali Z score 5.4). The sequence alignment compares GAG and CERV regions of Cer1 in *C*.*e* (*elegans*, N2 strain) with the corresponding regions in Cer1 elements present in five diverse, male-female species of *Caenorhabditis*: *C*.*n* (*nigoni*, JU1422 [134]); *C*.*r* (*remanei*, PX506 [135]); *C*.*z* (*zanzibari*, JU2190 [136]); *C*.*l* (*latens*, PX534 [137]); *C*.*i* (*inopinata*, TK-2017 [138]). Cer1 elements were identified from public databases as sequences that (1) had significant homology to the POL domain of *C*.*e* Cer1, (2) were flanked by direct repeats >100 base pairs, and (3) had a predicted tRNA-Pro primer binding sequence (TGGGGGCCG) adjacent to the 5’LTR, as is characteristic of the Cer1 family [24]. Cer1 chromosomal insertion sites, or Genbank identifiers for unassembled contigs, were as follows: *C*.*n* (insertion at LGX:20,441,949), *C*.*r* (Genbank: WUAV01000020.1), *C*.*z* (Genbank: UNPC02004551), *C*.*l* (Genbank: NIPN02000054.1), *C*.*i* (insertion at LGI:17980975). The complete alignment including the POL regions was used to generate the summary plot in Fig 2A. *C*. *elegans* CERV is the product of a spliced, five exon mRNA; exons 1–3 and exon 5 have the same reading frame as the CER1 polyprotein, and exon 4 has a different reading frame as described [24]. Splicing of CERV has not been examined in other *Caenorhabditis* species, but nucleotide alignments for each of the listed species showed consensus splice acceptor sequences similar to those in *C*. *elegans* preceding the predicted initiator ATG, and near each of the known exon boundaries. For example, the experimentally verified splice acceptor for exon 5 in *C*. *elegans* is TTTCAG [24], and the aligned sequences had either TTTCAG (*C*.*n*, *C*.*r*, *C*.*z* and *C*.*i)* or TTGCAG (C.l). The N-terminal half of CERV shares some peptide sequences with GAG, and includes a predicted Leucine-zipper dimerization motif (LKEKSEELMQKSQILVETTLKL; see also S4 Fig) and a monopartite nuclear localization sequence (KRKK; consensus K-(K/R)-X-(K/R). GAG contains a predicted Leucine-rich nuclear export signal ^440^LNALANRLRL^449^ in the form L-x(2,3)-[LIVFM]-x(2,3)-L-x-[LI] that is removed in the spliced CERV product [139].
(TIF)

S2 Fig Alignment of Cer1 polyproteins in *Caenorhabditis* species; amino acids 446–766. (TIF)

S3 Fig Alignment of Cer1 polyproteins in *Caenorhabditis* species; amino acids 1871–2272. A previous report described limited amino acid similarities between the ORF encoded by CERV^CTD^ and the Env protein of *Drosophila* Gypsy (Genbank AAK52059.1), and suggested that the ORF might be an envelope protein [19]. Using Clustal Omega [140], we identified 79/517 amino acids in the complete CERV protein that were shared with Gypsy Env, but only nine of these were present in three or more of the *Caenorhabditis* Cer1 elements. Those residues, numbered relative to the Cer1 polyprotein/CERV, are R1949/194, F1957/202, I1979/224, E2033/278, W2036/281, L2048/293, I2055/300, I2174/419, and Y2199/444.
(TIF)

S4 Fig Structural models for Cer1 GAG and CERV. **A.** Diagram comparing sizes and domains of GAG proteins from Ty3 [45]; HIV-1 [46]; and RSV [47] with the predicted GAG region of Cer1. For reference below, the CERV^NTD^ is aligned with Cer1 GAG to show the position of in-frame peptides common to both (exons 1–3) and the unique peptide (exon 4). Some retroviral GAG proteins contain short, disordered regions between MA, CA, and NC that might function in particle morphogenesis or GAG-RNA interactions, but which are removed from mature virions [141]. Cer1 GAG is predicted to have three intrinsically disordered regions acids (grey boxes: aa1-31, 263–358, 564–630) as scored by IUPRED server (https://iupred3.elte.hu/) using "long disorder" parameters [142], but it has not been determined whether these represent processing sites. **B.** Structural prediction for Cer1 GAG residues 387–546 generated with AlphaFold and colored as per the AlphaFold pLDDT table, a per-residue estimate of confidence on a scale of 0–100 [131]. The DALI server was used to compare this model against experimentally determined structures in the PDB as described for S1 Fig. The model showed the highest similarity to CA proteins from diverse retroviruses and endogenous LTR retrotransposons, and particularly the C-terminal half of CA which mediates subunit multimerization [143]. CA domains from RSV (PDB:3g29) and PEG10 (PDB:7lga) are shown in the middle panel for comparison, along with a superimposition of those structures with the Cer1 model (ChimeraX Matchmaker [144]). The table shows quantitative data for representative structural alignments with DALI Z-scores and root-mean-square deviations (RMSD) from backbone in Angstroms; DALI Z-scores above 2 usually correspond to similar folds [145]. **C**. AlphaFold structural prediction for the large region of Cer1 GAG preceding the CA-like domain, colored as per the pLDDT table [131]. The model shows three long, anti-parallel alpha-helices, the longest of which contains a predicted leucine zipper (LZ). The red brackets mark the boundaries of the three peptides (e1-e3) that are spliced in-frame to make the CERV^NTD^. The model was searched against the PDB as above, but did not appear to have significant similarity to known proteins, including retroviral MA proteins. **D.** AlphaFold structural prediction for CERV. CERV residues here and below are numbered with respect to the CERV protein, rather than the entire Cer1 polyprotein. The complete CERV protein (aa1-517) was used for the structural prediction, but for clarity the terminal unstructured regions (1–20 and 470–517) are hidden. Red bars correspond to exon boundaries. The H domain consists of long, anti-parallel alpha-helices shared in part with GAG, and does not appear to have significant similarity to known proteins. The G and M domains are described below and in S6 Fig. **E.** The AlphaFold structural prediction for the G domain of *C*.*e CERV* is shown at top left, colored arbitrarily to indicate alpha-helices (salmon) and beta-strands (green). Highly similar structural models for the G domain were obtained in independent modeling for each of the *Caenorhabditis* species aligned in S1 Fig, shown here at smaller scale for *C*. *zanzibari* and *C*. *inopinata*. The G domain consists of five alpha helices (α1-α5) surrounding a five-stranded parallel β sheet; α1 and α5 are on one side of the sheet, and α2–4 are on the opposite side. This basic structure is termed a Rossmann-type fold, and is a common structural motif in nucleotide-binding proteins [146]. Most of those proteins have conserved motifs associated with ATP or GTP hydrolysis, such as the Walker A motif or P-loop (phosphate binding loop) [147], but some structurally similar proteins of unknown function have been identified, such as pseudoGTPases [148]. The G domain of CERV lacks critical residues in the P-loop and other motifs (bottom right), indicating that the G domain is not predicted to function in GTP hydrolysis. The DALI server was used as above to search for structures in the PDB with similarity to the G domain, and matches were found to numerous and diverse nucleotide-binding proteins, here shown for human Rag GTPase (PDB: 6wj2-F; DALI Z-score 9.2). The G domain also showed structural similarity to a few proteins that are not predicted to be NTP-binding proteins. One of these, TSR1 (PDB: 5wwn-A; DALI Z-score 6.2), is a pre-rRNA-processing protein that appears to be an inactive, structural mimic of a GTPase [149]. A second example is the TIR domain of the plant *Arabidopsis* immune receptor RRS1 (PDB: 4c6t-A; DALI Z-score 9.7). The TIR (Toll-interleukin-1 receptor) domain is thought to mediate protein heterodimerization in response to pathogen infection [150].
(TIF)

S5 Fig Cer1 gRNA persistence after RNAi. For each experiment in this figure, worms were grown on empty-vector (control) bacteria until the stage indicated in brackets (see Fig 1A for staging). The worms were either processed immediately, transferred to a second plate of control bacteria, or transferred to a culture plate of bacteria with a dsRNA insert specific for the target gene. For example, "[A1] plus 6 hrs *gfp(RNAi)*" means that staged A1 animals on control bacteria were transferred to *gfp(RNAi)* feeding plates for 6 hrs before processing. Sets of 30–40 gonads were analyzed for each experiment; the panels show either single, representative images for a time point, or show multiple images at the same timepoint where the results varied appreciably. **A.** Comparison of *gfp-cerv* mRNA expression (strain WM638) in a control worm without RNAi and in a worm treated with *gfp(RNAi)* for 6 hrs; the gonads were hybridized with oligos specific for *gfp* (S3 Table). *gfp(RNAi)* markedly reduced the *gfp-cerv* mRNA signal by 4 hrs, and most of the signal was gone by 6 hrs as shown; additional time points analyzed (8 hrs, 10 hrs) appeared similar to 6 hrs. In all RNAi experiments, many of the few remaining cytoplasmic foci (arrows) were much larger than typical mRNA foci in control gonads but were not analyzed further. WM638 has *gfp* inserted at the 5’ end of *cerv*; this insertion disrupts CERV function such that gRNA is not exported (see S7 Fig). Thus, the *gfp* probe is expected to recognize nuclear signals from both *gfp*:*cerv* and *gfp*-containing *gRNA*, but can only recognize *gfp*:*cerv* in the cytoplasm. We conclude that RNAi is highly effective in removing cytoplasmic, spliced *gfp*:*cerv* mRNA, which is not associated with GAG. **B.** This experiment addresses whether RNAi can block newly synthesized gRNA from accumulating in the cytoplasm. L4 wild-type larvae, which have little detectable cytoplasmic gRNA, were placed for the indicated times on control bacteria, or on bacteria with a dsRNA insert specific for Cer1 reverse transcriptase [hereafter *gRNA(RNAi)*]. *gRNA(RNAi)* for 24 hrs caused a moderate decrease in cytoplasmic gRNA compared to control gonads, but *gRNA(RNAi)* for 48 hrs caused a much larger relative decrease. The projected images from the 48 hour control and RNAi gonads were indexed and scored blindly as one of four classes [++++ / +++ / ++ / +] based on the abundance of immunostained GAG particles. Control gonads were scored as [37.8% / 47.2% / 15% / 0%, n = 36], and *gRNA(RNAi)* gonads were scored as [6.7% / 37.8% / 40.0% / and 15.5%, n = 45]. The images shown for the 48 hr control and RNAi experiment (panel 2) represent the most common class for each (+++ and ++, respectively). Panel 3 is a single optical plane of the post-pachytene region of a 48 hr *gRNA(RNAi)* gonad, and shows that the few remaining gRNA foci colocalize with GAG; asterisks indicate nuclear foci of gRNA. We conclude (1) that *gRNA(RNAi)* does not block newly synthesized gRNA from accumulating, but substantially reduces the accumulation compared to controls without RNAi, and (2) most of the cytoplasmic gRNA remaining after RNAi is associated with GAG. **C.** This experiment addresses the effectiveness of *gRNA(RNAi)* in removing previously accumulated gRNA. RNAi-sensitive wild-type animals were compared with control, RNAi-resistant *rde-1(ne300)* mutant animals [151]. A1 adults, which have accumulated substantial amounts of cytoplasmic gRNA (see Fig 2E) were placed on *gRNA(RNAi)* bacteria for 48 hrs before processing. Gonads were analyzed by smFISH for gRNA, then photographed at identical exposures (shown in black/white for better resolution). Single images representing 10-micron Z-projections were generated for each gonad, and the images of the wildtype *and rde-1* gonads were scored blindly for the abundance of gRNA [++++ / +++ / ++ / +] as above. The images depict each of the four classes of gRNA abundance, and were taken from the RNAi-resistant *rde-1* animals exposed to *gRNA(RNAi)*. Classification of the *rde-1* and WT gonads is quantified at the bottom of the panel. The data suggests that *gRNA(RNAi)* lowers the level of gRNA in WT gonads relative to RNAi-resistant *rde-1* controls, but that a considerable amount gRNA remains. **D.** This experiment compares wild-type animals which were selected at the A1 stage, then divided into two populations which were fed for the indicated times with either empty-vector control bacteria or *gRNA(RNAi)* bacteria. Note the persistence of gRNA after *gRNA(RNAi)*, and the colocalization of gRNA with GAG.
(TIF)

S6 Fig Structural models of the CERV M domain. **A.** The model at left shows the AlphaFold structural prediction for the M domain of the CERV protein in *C*. *elegans* Cer1, with pLDDT coloring for confidence scores as above. The models at right show independent predictions using AlphaFold for M domains from Cer1 elements in each of the five *Caenorhabditis* species aligned in S3 Fig. Despite the low degree of sequence conservation in the M domain (S3 Fig), the models are closely similar with the principal difference being short beta-strands present in *C*.*e*, *C*.*r*, and *C*.*l*, but not in *C*.*n*, *C*.*z*, or *C*.*i*; models for each of the two groups are shown in superimposition and colored arbitrarily. **B.** The two top images show octamer models for the M domains of CERV from *C*.*e* and *C*.*l*, colored according to pLDDT scores. Space-filling models for each octamer (below) show the similar electrostatic potentials (red negative, blue positive; ChimeraX [132]). The M domains from each of the *Caenorhabditis* species are predicted to form similar ring-shaped multimers; rings form with a minimum of 5 subunits, and rings consisting of 5–8 subunits have high confidence scores. The inset at right is a high magnification ribbon view of the indicated 3 subunits, shown with arbitrary colors. The lines between the subunits represent residue contacts which are 3 Å or less and colored according to the AlphaFold pLDDT confidence scale (ChimeraX [132]. The asterisk indicates the S214 phosphorylation site described in the text.
(TIF)

S7 Fig N-terminal tags disrupt CERV function. **A**. Characterization of WM638, which has *gfp* inserted at the shared 5’ terminus of *cerv* and *gag*. The strain was expected to make GFP:CERV and GFP:GAG, but the live animals had very few GFP:GAG particles and did not make GFP:CERV rods. Panel 1 shows that WM638 expresses abundant *gfp-*containing mRNA in the core cytoplasm, as detected by smFISH with probes specific for *gfp*. By contrast, panel 2 shows that a gRNA-specific probe does not detect gRNA in the WM638 core, and that GAG is not expressed. Thus, the core contains spliced *gfp*:*cerv* mRNA, but does not contain gRNA (with unspliced *gfp*). Additional experiments showed that the WM638 animals do not make CERV rods. Panel 3 shows that immunostained CERV foci (red, mAbP3C6) are present in the WM638 germ nuclei, and that nearly all of these foci are coincident with GFP:CERV (green, anti-GFP). However, the WM638 nuclei usually do not have closely paired CERV foci as found in wild-type nuclei, but instead have single foci or dispersed, multiple foci. Panel 4 shows a 6-micron Z-projection of a field of WM638 germ nuclei stained for gRNA (red) and GFP (green). The inset at right shows that most of the GFP:CERV foci do not colocalize with nuclear gRNA. These combined results suggest that the N-terminal GFP tag disrupts CERV function such that gRNA is not exported and GFP:GAG is not expressed. **B.** Characterization of WM638/WT heterozygotes. Images are from heterozygous animals generated by crossing wild-type males into WM638 hermaphrodites. By contrast with the WM638 homozygotes, both fixed and immunostained heterozygotes showed GFP:CERV localization in foci, streaks, and rods (panel 1), and cytoplasmic gRNA was abundant in the core (panel 2). The heterozygotes made abundant GFP:GAG particles which were visible in live animals and by staining for GFP (panel 3), many of which colocalized with gRNA (panel 3). Thus, the heterozygotes appear to incorporate the GFP-tagged CERV and GAG proteins into the correct structures, presumably by association with the respective, untagged proteins. **C.** Characterization of JJ2698. Because GFP:CERV must be combined with an untagged copy of CERV to localize properly, this strain was built to be homozygous for both tagged and untagged copies of CERV (see Methods for details) The strain has the N2 copy of Cer1 plus a linked copy of Cer1 derived from the wild strain ED3046 in which CERV was tagged with GFP. Similar to WM638 heterozygotes, the homozygous JJ2698 animals had nuclear foci of GFP which colocalized with CERV and nuclear gRNA (panel 1, asterisks), they made GFP-containing GAG particles that colocalized with cytoplasmic gRNA (panel 2, arrowheads), and they made GFP-containing CERV rods (panel 3; 6-micron Z-projection).
(TIF)

S8 Fig Stage-dependent changes in nuclear gRNA in wild-type and *fog-2* mutant germ cells. **A.** Age-dependent decrease in nuclear gRNA in wild-type hermaphrodites. The images show pachytene germ nuclei in wild-type hermaphrodites at A1 or A4, stained for gRNA, GAG, and CERV as indicated. Fixed gonads from each population were dissected to create a population-specific identifying mark, then mixed together for immunostaining and photography; each channel was imaged at the same exposure used for the wild-type A1 animals. Asterisks mark nuclear foci of gRNA, and arrowheads mark representative foci of cytoplasmic gRNA; arrows indicate unusually bright foci of cytoplasmic gRNA in both samples that colocalize with GAG and likely represent aggregates of capsids. The boxed regions are shown at higher magnification in the insets at right, except that CERV is shown instead of GAG. Note that the level of nuclear gRNA (asterisks) decreases appreciably by A4. In the low magnification image of the A4 gonad, most germ nuclei appear to lack nuclear gRNA foci: This is an artifact of the sectioning plane; gRNA signals are present but reduced, and are typically visible in only a single optical plane. By contrast, the bright nuclear gRNA signals in the A1 gonad are visible in several optical planes. **B.** Age-dependent changes and effect of mating on nuclear gRNA in *fog-2* females. gRNA, GAG, and CERV as indicated are compared for wild-type A1 animals, unmated *fog-2* females from A1-A3, and an A4 *fog-2* female that was mated at the A3 stage. Calibration of signal intensities was as described for S8A. The level of nuclear gRNA and CERV (asterisks) in A1 *fog-2* animals appears comparable to wildtype, but decreases markedly by A3. However, mating for 24 hours restores the levels of nuclear gRNA and CERV. **C.** Mated-induced increase in CERV rods in *fog-2(q71)* mutant. The images compare rods in unmated A1 and A5 *fog-2* mutant gonads with rods in an A5 *fog-2* animal that was mated at A4; see Fig 5C for quantification). Note the large increase in the size of the gonad between A1 and A5, indicating that the gonad does not arrest development in unmated *fog-2* animals. **D.** Rod variations in mated wild-type animals. Most rods in older mated animals resemble those in younger animals, but with the following variations. The short arrow in panel 1 indicates a rod with a diameter of 0.5 microns, which is similar in size to rods in younger, unmated animals (see Fig 5D). However, a small percentage of rods (3/112) have abnormally large diameters; the long arrow indicates a rod with a diameter of 1.0 microns. Panel 2 shows an example of a large, hemicylinder of CERV with gRNA shown in red; similar hemicylinders were found in 3/318 germ nuclei. Panel 3 shows a group of germ nuclei with multiple rods. Panel 4 shows a 3D optical projection of a germ nucleus with adjacent, parallel rods (arrowheads); "X’s" visible in the background are 3D reference marks. Similar double rods occur in less than 1% of nuclei (1/112); see Fig 8B, panel 2 for TEM of a double rod. Note that the rods do not track the S-shaped LGIII (dotted line; identified by the nuclear foci of gRNA). Bars = 2.0 microns.
(TIF)

S9 Fig TEM of nuclear fibrils. **A.** Measurements of individual fibril lengths (n = 150) taken from TEM micrographs; graphing with GraphPad Prism software version 9.5. **B.** High magnification of three long fibrils (344, 477, and 497 nm); sample as in Fig 7D. Note the numerous striations (arrows) which are approximately orthogonal to the long axis of the fibril. **C.** Images of sequential, 70 nm thick Z-sections through a curved CERV rod. The perimeter of the rod has an electron-lucent margin (white arrowheads), and electron-dense fibrils are near the perimeter and inside the rod. The arrows at 140nm and 210nm indicate a few electron-dense fibrils which are associated with the nucleolus, but do not appear to have become incorporated into the rod.
(TIF)
