# Supplementary material for: The CERV protein of Cer1, a C. elegans LTR retrotransposon, is required for nuclear export of viral genomic RNA and can form giant nuclear rods
Source: PLoS Genet. 2023 Jun 29;19(6):e1010804. doi: 10.1371/journal.pgen.1010804 (PMC10309623; doi:10.1371/journal.pgen.1010804)
Supplement: S1 Table — (DOCX) [file pgen.1010804.s001.docx]

**S1 Table. Strain list**

| **strain** | **genotype and description** |
| --- | --- |
| N2 | Wildtype laboratory strain of *C. elegans* |
| CB4507 | wild strain of *C. elegans* |
| CB4856 | wild strain of *C. elegans* |
| CB4932 | wild strain of *C. elegans* |
| CX11307 | wild strain of *C. elegans* |
| ED3046 | wild strain of *C. elegans* |
| EG4946 | wild strain of *C. elegans* |
| JU406 | wild strain of *C. elegans* |
| MY16 | wild strain of *C. elegans* |
| MY23 | wild strain of *C. elegans* |
| CB1370 | *daf-2(e1370)* |
| CB4108 | *fog-2(q71)* |
| MT1522 | *ced-3(n717)* |
| YL206 | *nst-1p::nst-1::*GFP*::nst-1 3'UTR + unc-119(+)* |
| **this study** |  |
| JJ2506 | *zuIs242(nmy-2::*PGL-1::GFP::*pie-1 3'UTR); zuIs256(cer1*Protease::GFP*::pie-1 3'UTR); unc-119(ed3).* |
| JJ2669 | Cer1[ED3046, LGIII*;* GFP:CERV/GAG] |
| JJ2698 | *glo-2(zu455);* Cer1[N2, LGIII: -0.04 ] *unc-32(e189)* Cer1[ED3046, LGIII; GFP:CERV/GAG] |
| JJ2699 | *cer1(zu526*) CERV[T212A,S214A,T216A]) |
| JJ2700 | *cer1(zu527)* CERV [C193S, C195S, C200S]) |
| JJ2704 | *cer1(zu531)* CERV [R194A]) |
| JJ2705 | *cer1(zu532)* CERV [T212A, S214A]) |
| JJ2706 | *cer1(zu533)* CERV [S214A]) |
| WM638 | *cer1(ne4927)* GFP:CERV/GAG |
| WM743 | *cer1(ne4967)* GAG:GFP |
| WM744 | *cer1(ne4976)* GFP:APEX2:CERV/GAG |
| WM746 | *cer1(ne4881*) CERV(STOP) exon4 |
| WM790 | *cer1(ne4977)* GFP::APEX2::CERV/GAG |
| WM894 | *cer1(ne4928; ne4968)* 3XFLAG:CERV/GAG + GAG (STOP) |
| WM895 | *cer1(ne4975)* GAG:3XFLAG |
| WM903 | *cer1(4928)* 3XFLAG:CERV/GAG |
|  |  |
