# Supplementary material for: The CERV protein of Cer1, a C. elegans LTR retrotransposon, is required for nuclear export of viral genomic RNA and can form giant nuclear rods
Source: PLoS Genet. 2023 Jun 29;19(6):e1010804. doi: 10.1371/journal.pgen.1010804 (PMC10309623; doi:10.1371/journal.pgen.1010804)
Supplement: S2 Table — (DOCX) [file pgen.1010804.s002.docx]

**S2 Table. oligo sequences used for smFISH**

| ***gRNA*** *5' to 3'* | ***gfp*** *5' to 3'* |
| --- | --- |
| ttcttacactaccagtcaca | aaaagttcttctcctttact |
| tttgtgcgagcatcatttga | caagaattgggacaactcca |
| actccagaattgctacttgt | cccattaacatcaccatcta |
| ccgatgattctcatcttcta | cctctccactgacagaaaat |
| cacgattgatctggcttacg | gtaagttttccgtatgttgc |
| ttcaggagaaactctgcgga | gtagttttccagtagtgcaa |
| agcgaagatgcatgtcagtg | acaagtgttggccatggaac |
| gcagccttgtttggagaaaa | ggtatctcgagaagcattga |
| tagttttccaactttgctct | tcatgccgtttcatatgatc |
| caccaaaacatatgccatcc | gggcatggcactcttgaaaa |
| ttcttcggacaattccaagc | ttctttcctgtacataacct |
| gatttttccgatttcaccat | gttcccgtcatctttgaaaa |
| caattttctcccaggtattt | tgacttcagcacgtgtcttg |
| cacatgtctcattgttggtg | taacaagggtatcaccttca |
| ccttttttcaaggacagcat | ataccttttaactcgattct |
| ctctcttaagatgctcgcaa | gtgtccaagaatgtttccat |
| agtatatcaggtagctctgt | gtgagttatagttgtattcc |
| ctcagtattcttgagcactc | gtctgccatgatgtatacat |
| tctttcagtaatggtgtgcg | ctttgattccattcttttgt |
| gccaatagaactttctgtgc | ccatcttcaatgttgtgtct |
| agacattttgcacaggttct | atggtctgctagttgaacgc |
| gcccatctttcaacaaaagc | cgccaattggagtattttgt |
| attaatgccaacggcatctt | gtctggtaaaaggacagggc |
| tttaggacattgagctccta | aagggcagattgtgtggaca |
| tgcaaaatgtgcttccgttt | tcttttcgttgggatctttc |
|  | tcaagaaggaccatgtggtc |
|  | aatcccagcagctgttacaa |
|  | tatagttcatccatgccatg |
