# Supplementary material for: The CERV protein of Cer1, a C. elegans LTR retrotransposon, is required for nuclear export of viral genomic RNA and can form giant nuclear rods
Source: PLoS Genet. 2023 Jun 29;19(6):e1010804. doi: 10.1371/journal.pgen.1010804 (PMC10309623; doi:10.1371/journal.pgen.1010804)
Supplement: S3 Table — (DOCX) [file pgen.1010804.s003.docx]

**S3 Table. CRISPR gene constructions**

| **Strain** | **Guide** | **Donor** |
| --- | --- | --- |
| JJ2699 | 5'ctgagttggtgatggcgtat | 5'gtatgttcaatatgactcttgtccctgaagctcatgctccagctccagcacagctttatcgaatgtactgcattatggagaaatc |
| JJ2700 | 5'tacaaggcttcagagcacat | 5'aattcttgtttcagttgtcgatatatctcacgatcagctctgaagccttcaatgttcaatatgactcttgtccctgaagcccata |
| JJ2704 | 5'tacaaggcttcagagcacat | 5'aattcttgtttcagttgtcgatatatctgcgcttgtgctctgaagccttgtatgttcaatatgactcttgtccctgaagcccata |
| JJ2705 | 5'ctgagttggtgatggcgtat | 5'gtatgttcaatatgactcttgtccctgaagctcatgctccagctccaactcagctttatcgaatgtactgcattatggagaaatc |
| JJ2706 | 5'ctgagttggtgatggcgtat | 5'gtatgttcaatatgactcttgtccctgaagctcatacgccagctccaactcagctttatcgaatgtactgcattatggagaaatc |
| WM638* | 5'ctgaagaatggaggtgaacg | primers:  5'acgaatttatttttgtttcagtgaaatctgaagatccggagggagtgga  5'ctcgatgatcctccttcagtatcctgtccctcgttcacctcagaacctccgccacc |
| WM743* | 5'gatgtattttcatttttctt | primers:  5'cgaaatggggcacattgcttggaattgtccgaagaagaacgagaacagtaaaggagaagaacttttc  5'ctacttttgccaccggagcttccttttctgatgtagatcctttgtatagttcatccatg |
| WM744** | 5'gaactatacaaaggtggcgg | primers:  5'ctgggattacacatggcatggatgaactatacaaa  5'gatcctccttcagtatcctgtccctcgttcacctcttgcaatgcatcagcgaatccaag |
| WM746 | 5'tcatcaaaatattcagatca | 5'aacattgaacaaaagaatcatcaaaatattcagatcatggatactagatgcacaacatcgagctcaagaatgaatacac |
| WM790 | 5'atctgaagatccggagggag;  tcaggatcaggatcagctat | 5'aacgaatttatttttgtttcagtgaaatctgaagatgggaaaatcttatccaacagtttctgctgatta |
| WM894 | 5'tggtttccaggtgagaaaag | 5'accacggagaagacattcgaaaatggtttccaggtgagatgaacatgaagtaaaaaagaaggatgtgattaaacatatt |
| WM895 | 5'gatgtattttcatttttctt | 5'tgtaacgaaatggggcacattgcttggaattgtccgaagaaaaatgaagattacaaagaccatgatggtgactataaggatcatgatattgactataaagacgatgacgataagaatacatcagaaaaggaagctccggtggcaaaagtagag |
| WM903 | 5'ctgaagaatggaggtgaacg | 5'tttatttttgtttcagtgaaatctgaagaatggattacaaagaccatgatggtgactataaggatcatgatattgactataaagacgatgacgataaggaggtgaacgagggacaggatactgaaggaggatcatcgag |

*Donor sequence amplified from *gfp* plasmid B2233; a gift from Masaki Shirayama

**Donor sequence amplified from *apex2* plasmid pCCM959; a gift from Daniel Durning
