# Supplementary figures and images for: The CERV protein of Cer1, a C. elegans LTR retrotransposon, is required for nuclear export of viral genomic RNA and can form giant nuclear rods
Source: PLoS Genet. 2023 Jun 29;19(6):e1010804. doi: 10.1371/journal.pgen.1010804 (PMC10309623; doi:10.1371/journal.pgen.1010804)

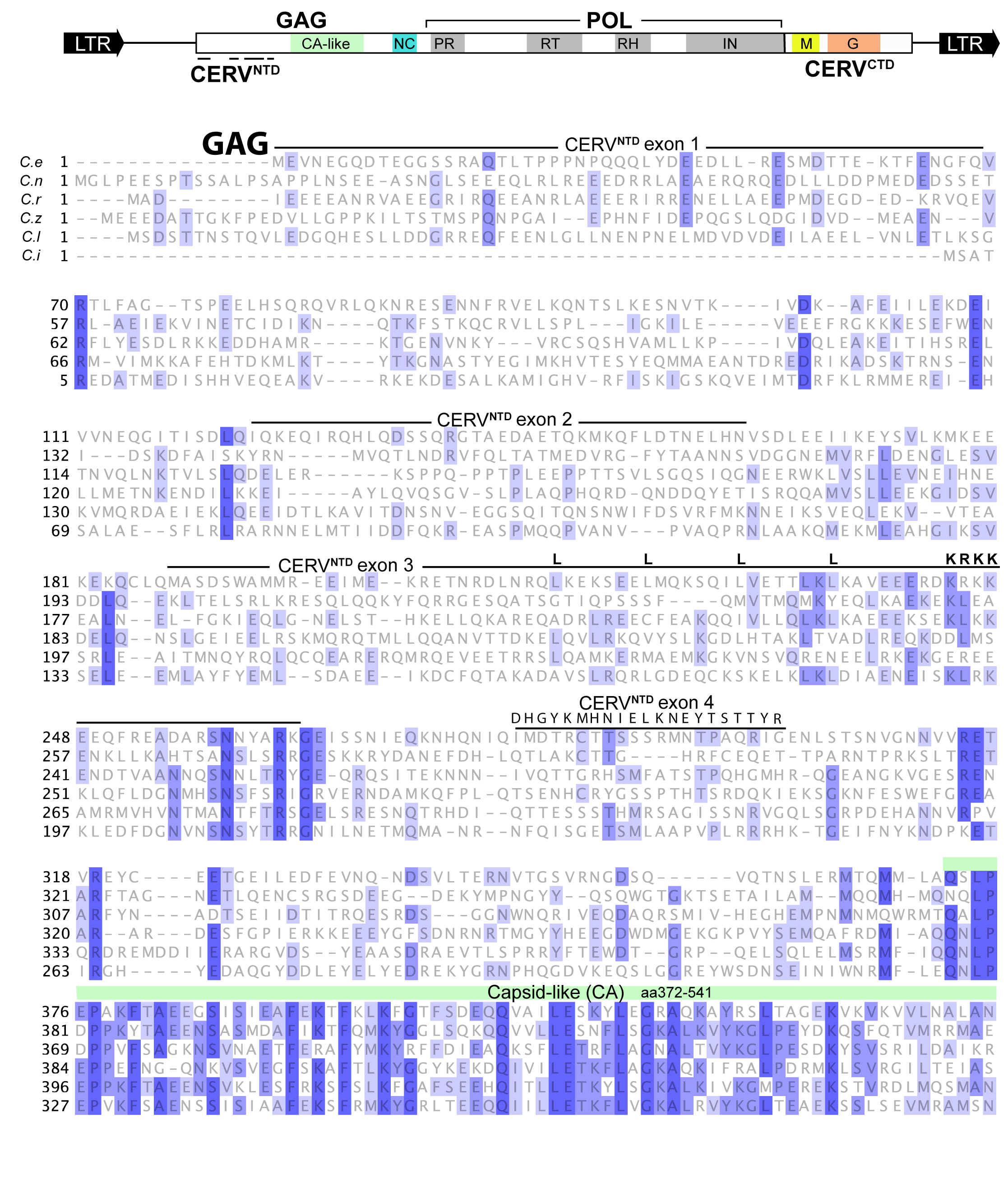

Supplement: S1 Fig — The diagram at top summarizes protein domains in Cer1 as described previously [21,24] and extended here (see below), plus an additional capsid-like (CA-like) domain in GAG. M and G subdomains of CERV are indicated and described in the text. The sizes shown for the various POL domains were updated as per InterPro protein family classifications (http://www.ebi.ac.uk/interpro/) [133]: protease (PR, aa735-859, IPR021109), reverse transcriptase (RT, aa1052-1231, IPR000477 plus 1241–1326, IPR043128), and ribonuclease H (RH, aa1327-1429, IPR041373). A previously described integrase (IN) domain in Cer1 (approximately aa1546-1603, IPR041588 plus aa1616-1774, IPR001584) was extended here to aa1873 based on structural prediction: AlphaFold and ColabFold [48,49] were used to generate a structural model for the extended region, which was compared with structures in the Protein Database (PDB) using the Dali server [51]. This analysis showed that the extension had structural similarity to integrase proteins from retroviruses such as Foamy Virus (PDB ID: 5frn-A; Dali Z score 6.8) and Rous Sarcoma Virus (PDB ID:1c0m-B; Dali Z score 5.4). The sequence alignment compares GAG and CERV regions of Cer1 in C.e (elegans, N2 strain) with the corresponding regions in Cer1 elements present in five diverse, male-female species of Caenorhabditis: C.n (nigoni, JU1422 [134]); C.r (remanei, PX506 [135]); C.z (zanzibari, JU2190 [136]); C.l (latens, PX534 [137]); C.i (inopinata, TK-2017 [138]). Cer1 elements were identified from public databases as sequences that (1) had significant homology to the POL domain of C.e Cer1, (2) were flanked by direct repeats >100 base pairs, and (3) had a predicted tRNA-Pro primer binding sequence (TGGGGGCCG) adjacent to the 5’LTR, as is characteristic of the Cer1 family [24]. Cer1 chromosomal insertion sites, or Genbank identifiers for unassembled contigs, were as follows: C.n (insertion at LGX:20,441,949), C.r (Genbank: WUAV01000020.1), C.z (Genbank: UNPC02004551), [file pgen.1010804.s008.tif]

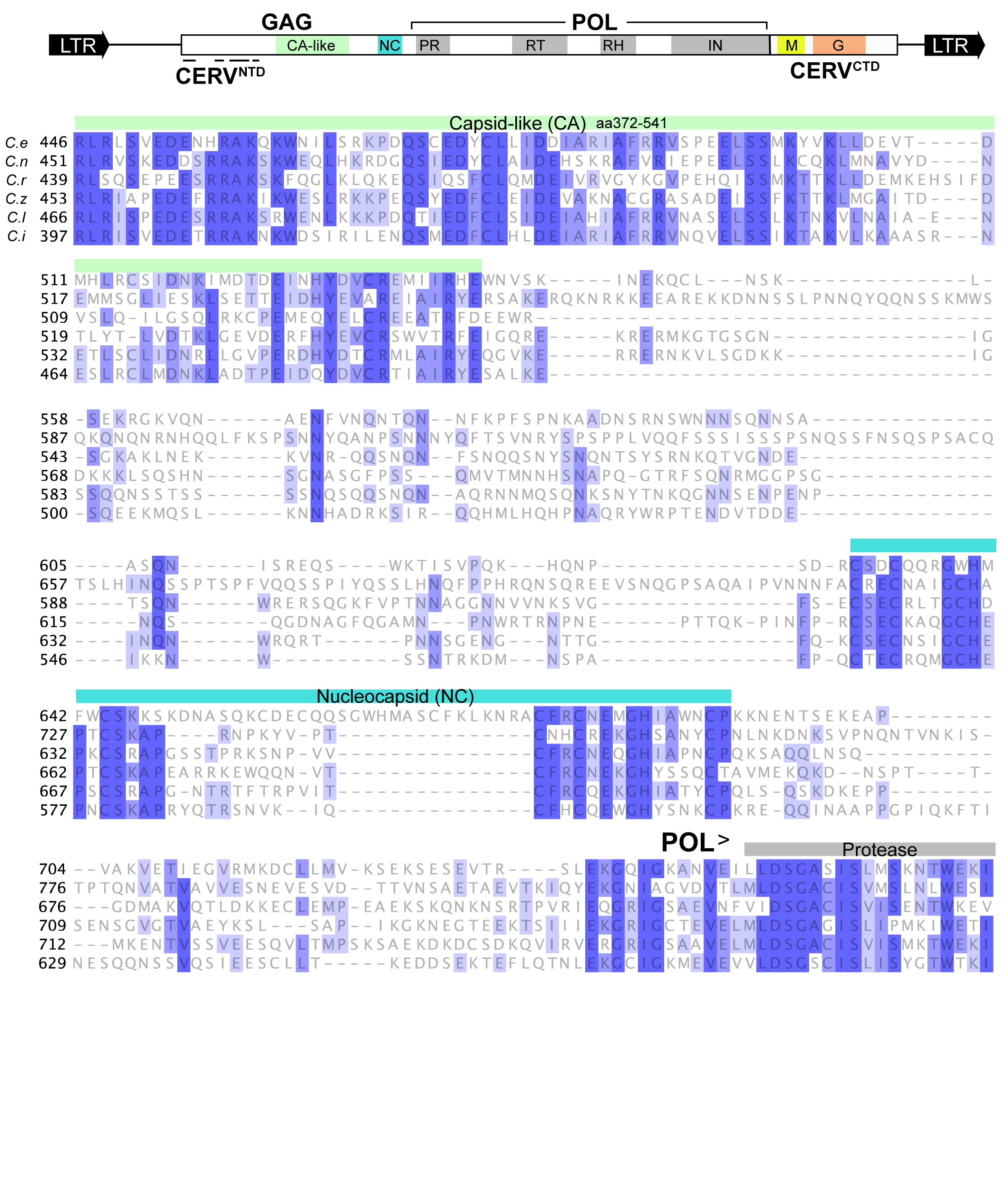

Supplement: S2 Fig — (TIF) [file pgen.1010804.s009.tif]

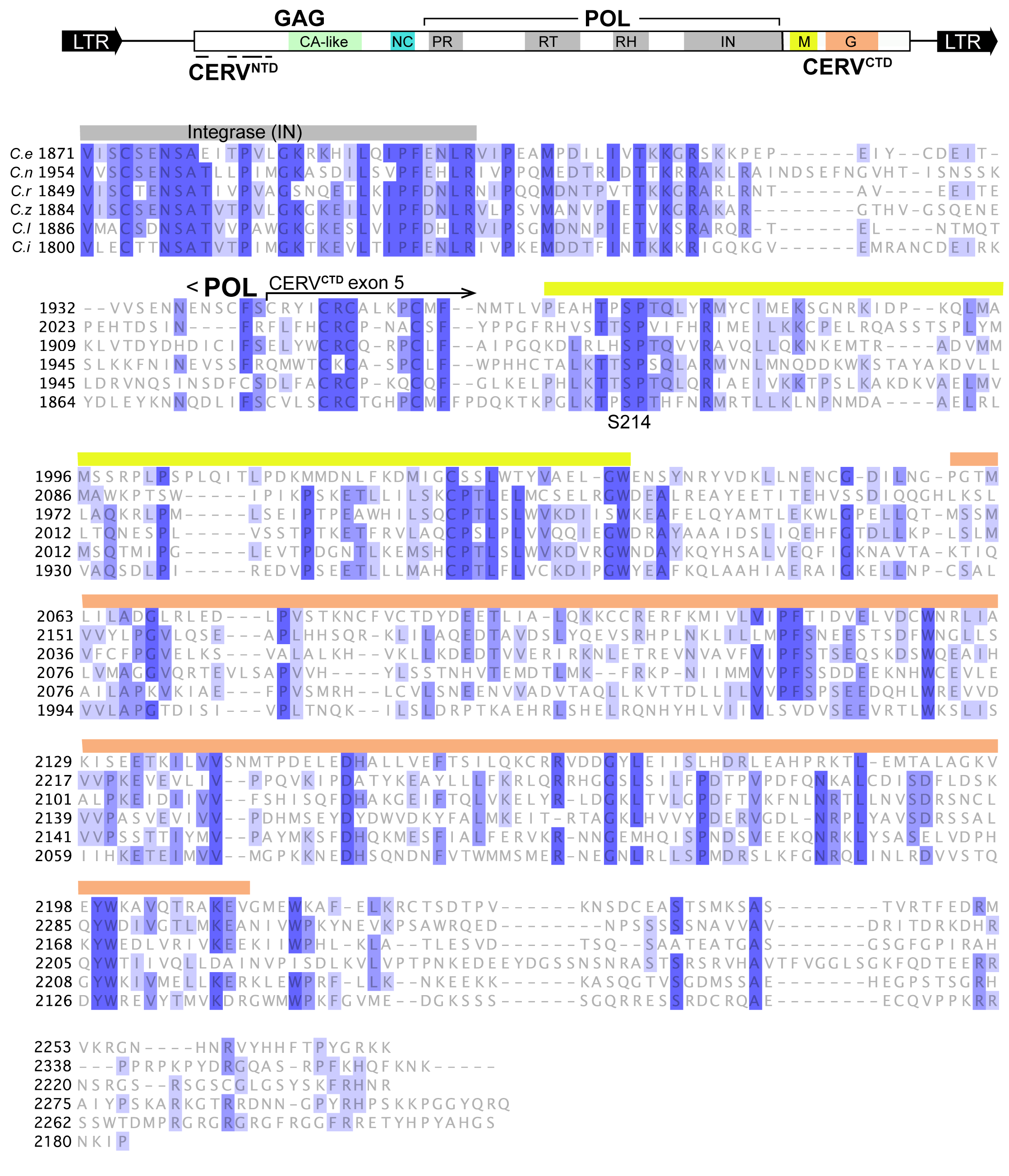

Supplement: S3 Fig — A previous report described limited amino acid similarities between the ORF encoded by CERVCTD and the Env protein of Drosophila Gypsy (Genbank AAK52059.1), and suggested that the ORF might be an envelope protein [19]. Using Clustal Omega [140], we identified 79/517 amino acids in the complete CERV protein that were shared with Gypsy Env, but only nine of these were present in three or more of the Caenorhabditis Cer1 elements. Those residues, numbered relative to the Cer1 polyprotein/CERV, are R1949/194, F1957/202, I1979/224, E2033/278, W2036/281, L2048/293, I2055/300, I2174/419, and Y2199/444. (TIF) [file pgen.1010804.s010.tif]

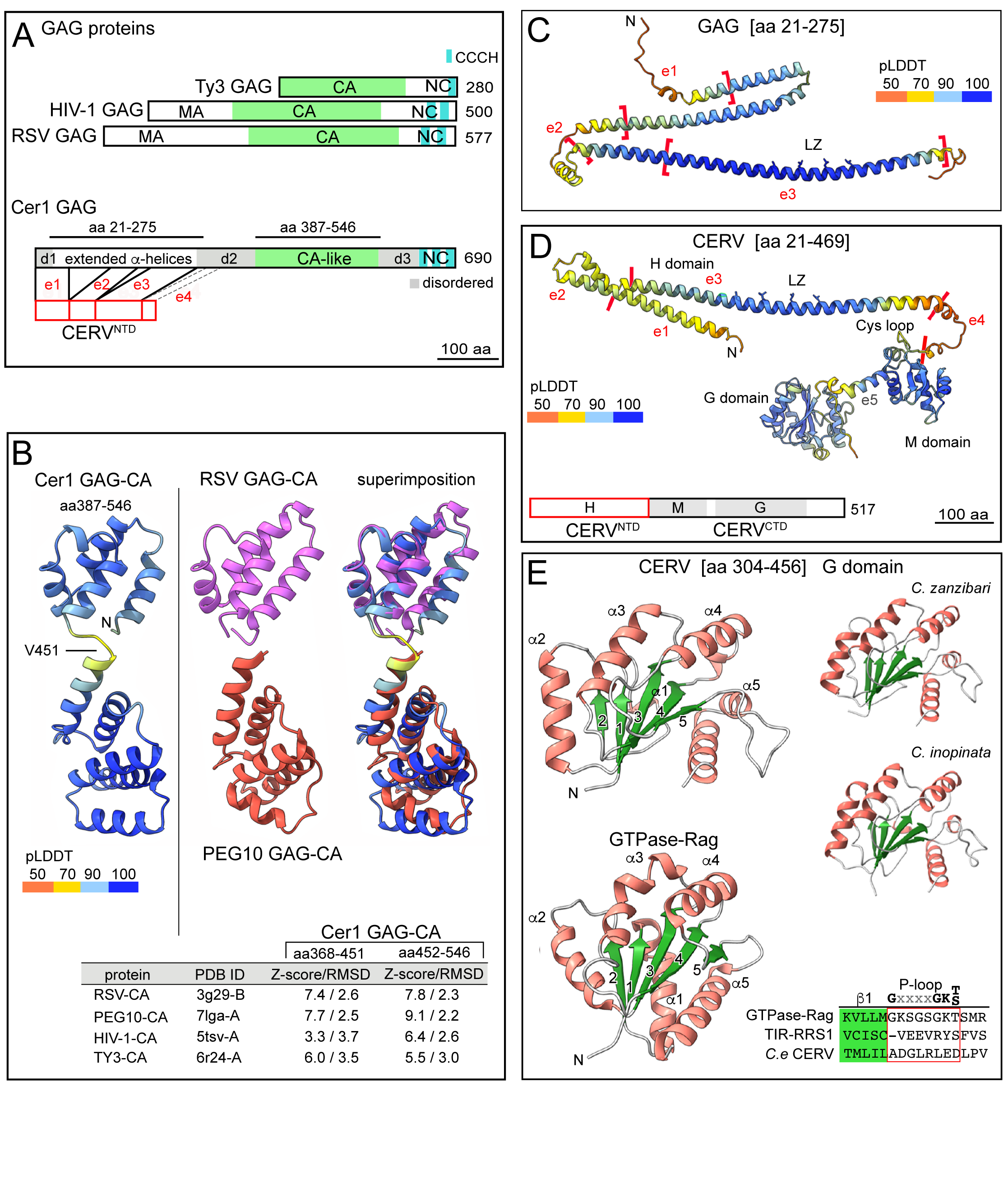

Supplement: S4 Fig — A. Diagram comparing sizes and domains of GAG proteins from Ty3 [45]; HIV-1 [46]; and RSV [47] with the predicted GAG region of Cer1. For reference below, the CERVNTD is aligned with Cer1 GAG to show the position of in-frame peptides common to both (exons 1–3) and the unique peptide (exon 4). Some retroviral GAG proteins contain short, disordered regions between MA, CA, and NC that might function in particle morphogenesis or GAG-RNA interactions, but which are removed from mature virions [141]. Cer1 GAG is predicted to have three intrinsically disordered regions acids (grey boxes: aa1-31, 263–358, 564–630) as scored by IUPRED server (https://iupred3.elte.hu/) using "long disorder" parameters [142], but it has not been determined whether these represent processing sites. B. Structural prediction for Cer1 GAG residues 387–546 generated with AlphaFold and colored as per the AlphaFold pLDDT table, a per-residue estimate of confidence on a scale of 0–100 [131]. The DALI server was used to compare this model against experimentally determined structures in the PDB as described for S1 Fig. The model showed the highest similarity to CA proteins from diverse retroviruses and endogenous LTR retrotransposons, and particularly the C-terminal half of CA which mediates subunit multimerization [143]. CA domains from RSV (PDB:3g29) and PEG10 (PDB:7lga) are shown in the middle panel for comparison, along with a superimposition of those structures with the Cer1 model (ChimeraX Matchmaker [144]). The table shows quantitative data for representative structural alignments with DALI Z-scores and root-mean-square deviations (RMSD) from backbone in Angstroms; DALI Z-scores above 2 usually correspond to similar folds [145]. C. AlphaFold structural prediction for the large region of Cer1 GAG preceding the CA-like domain, colored as per the pLDDT table [131]. The model shows three long, anti-parallel alpha-helices, the longest of which contains a predicted leucine zipper (LZ). The red brackets [file pgen.1010804.s011.tif]

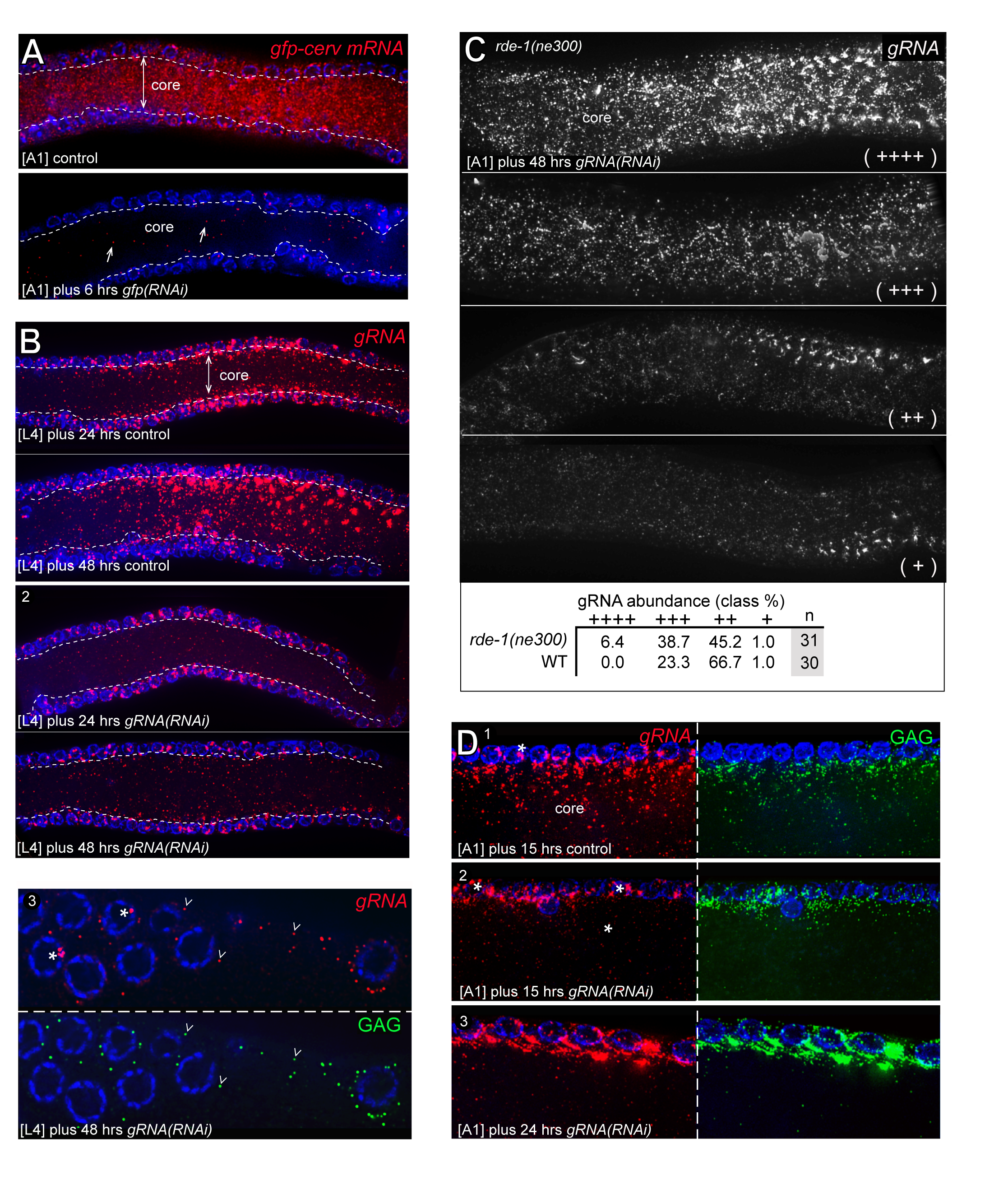

Supplement: S5 Fig — For each experiment in this figure, worms were grown on empty-vector (control) bacteria until the stage indicated in brackets (see Fig 1A for staging). The worms were either processed immediately, transferred to a second plate of control bacteria, or transferred to a culture plate of bacteria with a dsRNA insert specific for the target gene. For example, "[A1] plus 6 hrs gfp(RNAi)" means that staged A1 animals on control bacteria were transferred to gfp(RNAi) feeding plates for 6 hrs before processing. Sets of 30–40 gonads were analyzed for each experiment; the panels show either single, representative images for a time point, or show multiple images at the same timepoint where the results varied appreciably. A. Comparison of gfp-cerv mRNA expression (strain WM638) in a control worm without RNAi and in a worm treated with gfp(RNAi) for 6 hrs; the gonads were hybridized with oligos specific for gfp (S3 Table). gfp(RNAi) markedly reduced the gfp-cerv mRNA signal by 4 hrs, and most of the signal was gone by 6 hrs as shown; additional time points analyzed (8 hrs, 10 hrs) appeared similar to 6 hrs. In all RNAi experiments, many of the few remaining cytoplasmic foci (arrows) were much larger than typical mRNA foci in control gonads but were not analyzed further. WM638 has gfp inserted at the 5’ end of cerv; this insertion disrupts CERV function such that gRNA is not exported (see S7 Fig). Thus, the gfp probe is expected to recognize nuclear signals from both gfp:cerv and gfp-containing gRNA, but can only recognize gfp:cerv in the cytoplasm. We conclude that RNAi is highly effective in removing cytoplasmic, spliced gfp:cerv mRNA, which is not associated with GAG. B. This experiment addresses whether RNAi can block newly synthesized gRNA from accumulating in the cytoplasm. L4 wild-type larvae, which have little detectable cytoplasmic gRNA, were placed for the indicated times on control bacteria, or on bacteria with a dsRNA insert specific for Cer1 reverse transcriptase [her [file pgen.1010804.s012.tif]

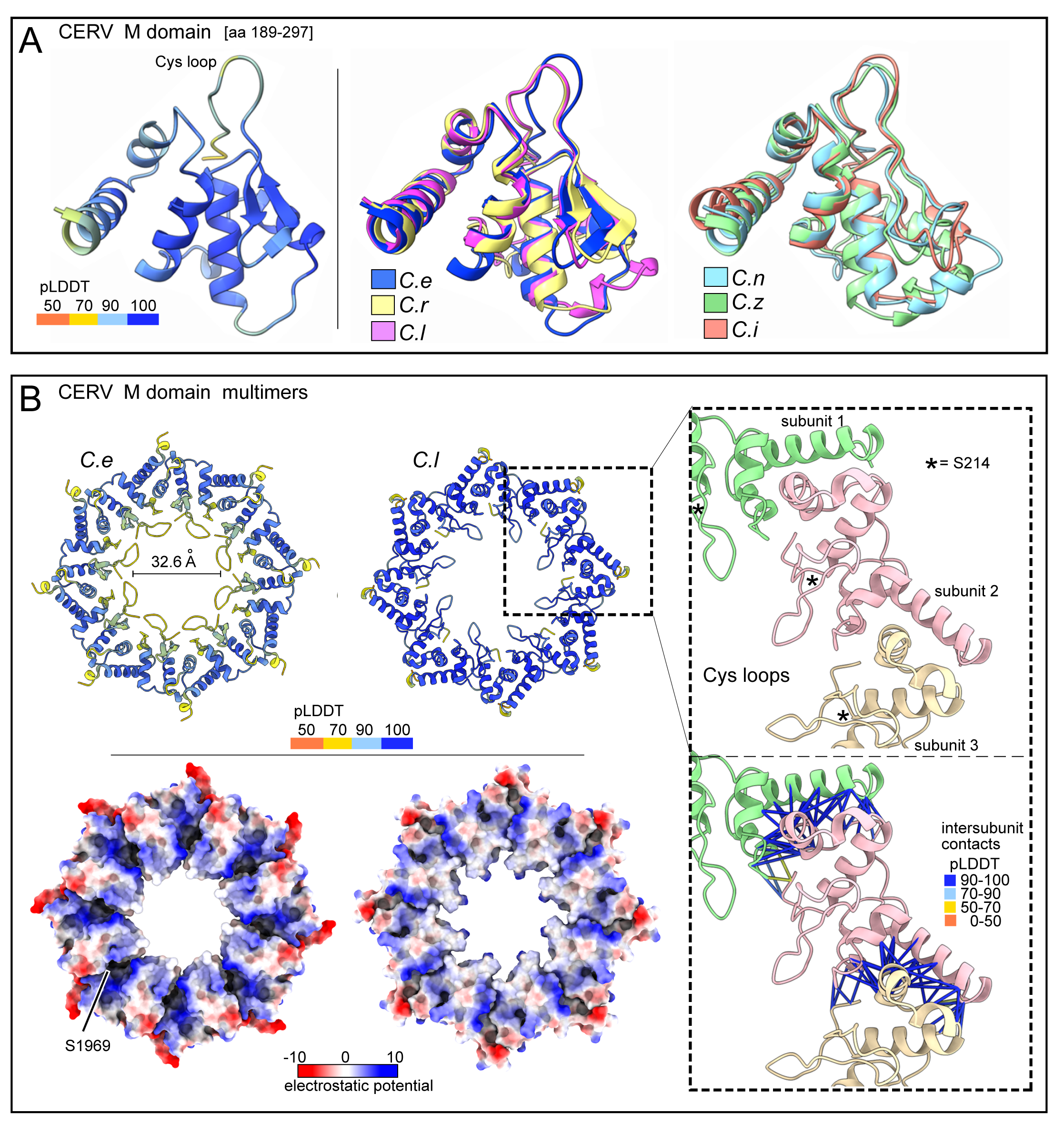

Supplement: S6 Fig — A. The model at left shows the AlphaFold structural prediction for the M domain of the CERV protein in C. elegans Cer1, with pLDDT coloring for confidence scores as above. The models at right show independent predictions using AlphaFold for M domains from Cer1 elements in each of the five Caenorhabditis species aligned in S3 Fig. Despite the low degree of sequence conservation in the M domain (S3 Fig), the models are closely similar with the principal difference being short beta-strands present in C.e, C.r, and C.l, but not in C.n, C.z, or C.i; models for each of the two groups are shown in superimposition and colored arbitrarily. B. The two top images show octamer models for the M domains of CERV from C.e and C.l, colored according to pLDDT scores. Space-filling models for each octamer (below) show the similar electrostatic potentials (red negative, blue positive; ChimeraX [132]). The M domains from each of the Caenorhabditis species are predicted to form similar ring-shaped multimers; rings form with a minimum of 5 subunits, and rings consisting of 5–8 subunits have high confidence scores. The inset at right is a high magnification ribbon view of the indicated 3 subunits, shown with arbitrary colors. The lines between the subunits represent residue contacts which are 3 Å or less and colored according to the AlphaFold pLDDT confidence scale (ChimeraX [132]. The asterisk indicates the S214 phosphorylation site described in the text. (TIF) [file pgen.1010804.s013.tif]

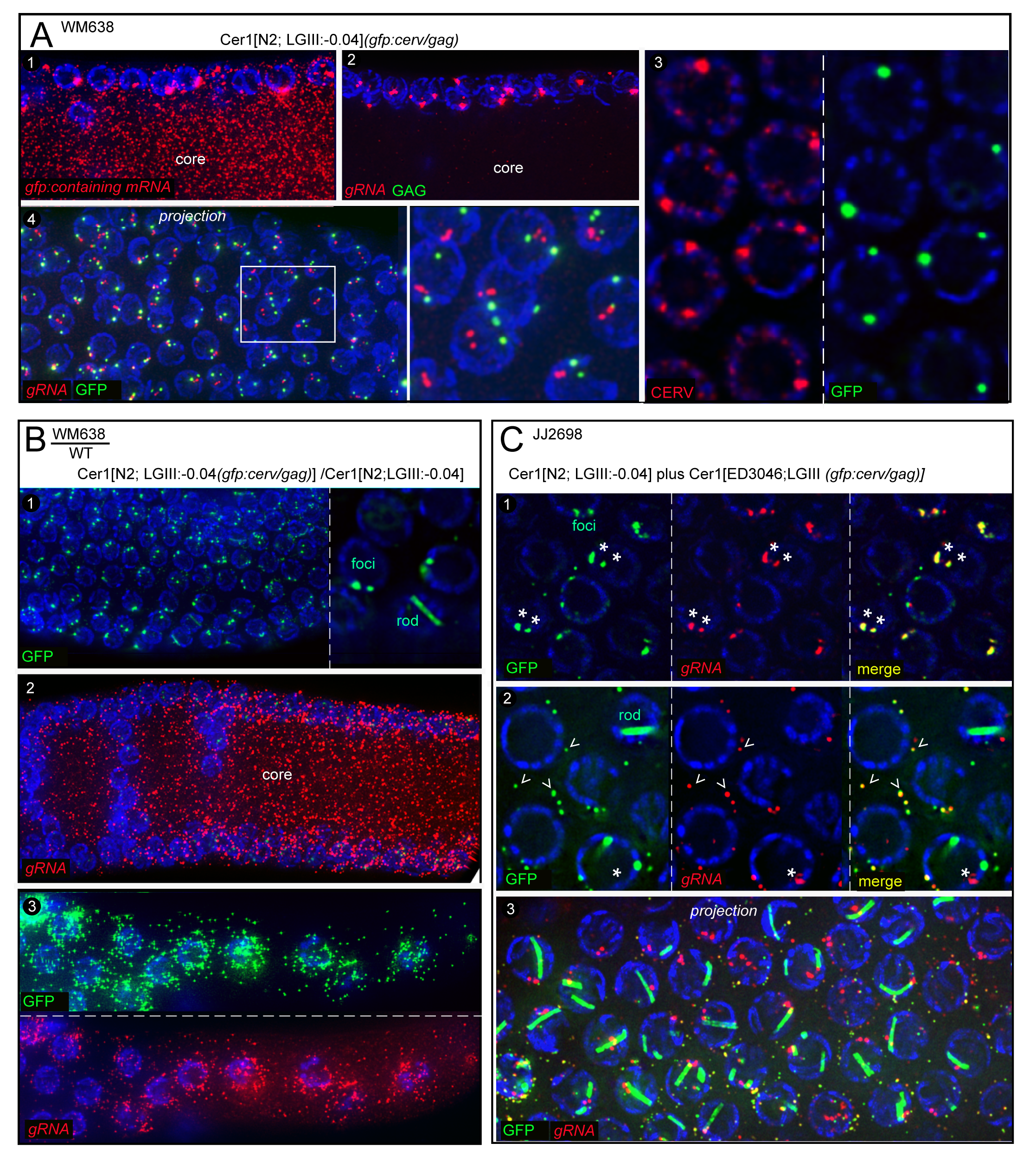

Supplement: S7 Fig — A. Characterization of WM638, which has gfp inserted at the shared 5’ terminus of cerv and gag. The strain was expected to make GFP:CERV and GFP:GAG, but the live animals had very few GFP:GAG particles and did not make GFP:CERV rods. Panel 1 shows that WM638 expresses abundant gfp-containing mRNA in the core cytoplasm, as detected by smFISH with probes specific for gfp. By contrast, panel 2 shows that a gRNA-specific probe does not detect gRNA in the WM638 core, and that GAG is not expressed. Thus, the core contains spliced gfp:cerv mRNA, but does not contain gRNA (with unspliced gfp). Additional experiments showed that the WM638 animals do not make CERV rods. Panel 3 shows that immunostained CERV foci (red, mAbP3C6) are present in the WM638 germ nuclei, and that nearly all of these foci are coincident with GFP:CERV (green, anti-GFP). However, the WM638 nuclei usually do not have closely paired CERV foci as found in wild-type nuclei, but instead have single foci or dispersed, multiple foci. Panel 4 shows a 6-micron Z-projection of a field of WM638 germ nuclei stained for gRNA (red) and GFP (green). The inset at right shows that most of the GFP:CERV foci do not colocalize with nuclear gRNA. These combined results suggest that the N-terminal GFP tag disrupts CERV function such that gRNA is not exported and GFP:GAG is not expressed. B. Characterization of WM638/WT heterozygotes. Images are from heterozygous animals generated by crossing wild-type males into WM638 hermaphrodites. By contrast with the WM638 homozygotes, both fixed and immunostained heterozygotes showed GFP:CERV localization in foci, streaks, and rods (panel 1), and cytoplasmic gRNA was abundant in the core (panel 2). The heterozygotes made abundant GFP:GAG particles which were visible in live animals and by staining for GFP (panel 3), many of which colocalized with gRNA (panel 3). Thus, the heterozygotes appear to incorporate the GFP-tagged CERV and GAG proteins into the correct structures, presumably by [file pgen.1010804.s014.tif]

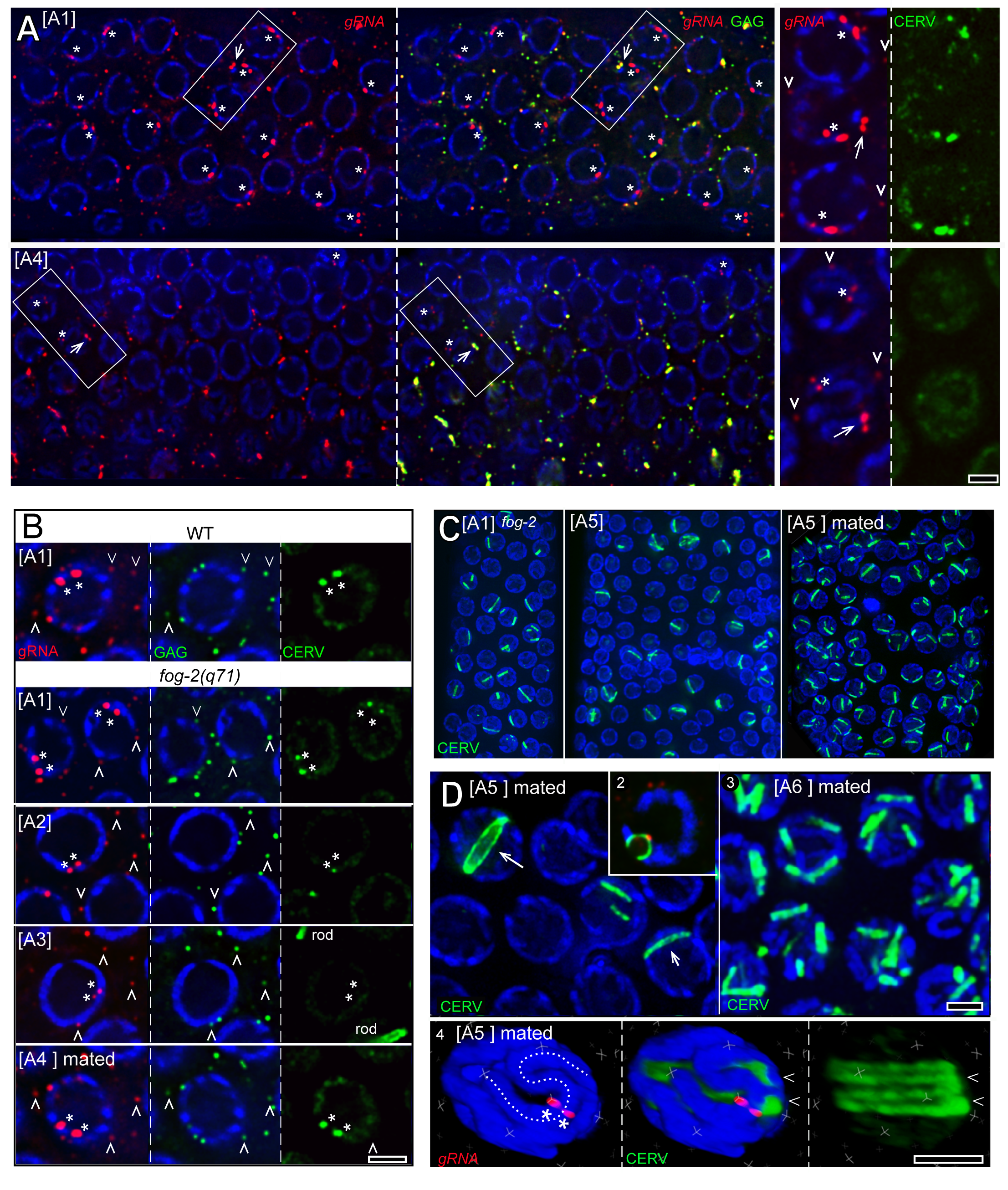

Supplement: S8 Fig — A. Age-dependent decrease in nuclear gRNA in wild-type hermaphrodites. The images show pachytene germ nuclei in wild-type hermaphrodites at A1 or A4, stained for gRNA, GAG, and CERV as indicated. Fixed gonads from each population were dissected to create a population-specific identifying mark, then mixed together for immunostaining and photography; each channel was imaged at the same exposure used for the wild-type A1 animals. Asterisks mark nuclear foci of gRNA, and arrowheads mark representative foci of cytoplasmic gRNA; arrows indicate unusually bright foci of cytoplasmic gRNA in both samples that colocalize with GAG and likely represent aggregates of capsids. The boxed regions are shown at higher magnification in the insets at right, except that CERV is shown instead of GAG. Note that the level of nuclear gRNA (asterisks) decreases appreciably by A4. In the low magnification image of the A4 gonad, most germ nuclei appear to lack nuclear gRNA foci: This is an artifact of the sectioning plane; gRNA signals are present but reduced, and are typically visible in only a single optical plane. By contrast, the bright nuclear gRNA signals in the A1 gonad are visible in several optical planes. B. Age-dependent changes and effect of mating on nuclear gRNA in fog-2 females. gRNA, GAG, and CERV as indicated are compared for wild-type A1 animals, unmated fog-2 females from A1-A3, and an A4 fog-2 female that was mated at the A3 stage. Calibration of signal intensities was as described for S8A. The level of nuclear gRNA and CERV (asterisks) in A1 fog-2 animals appears comparable to wildtype, but decreases markedly by A3. However, mating for 24 hours restores the levels of nuclear gRNA and CERV. C. Mated-induced increase in CERV rods in fog-2(q71) mutant. The images compare rods in unmated A1 and A5 fog-2 mutant gonads with rods in an A5 fog-2 animal that was mated at A4; see Fig 5C for quantification). Note the large increase in the size of the gonad between A1 and A5, indicati [file pgen.1010804.s015.tif]

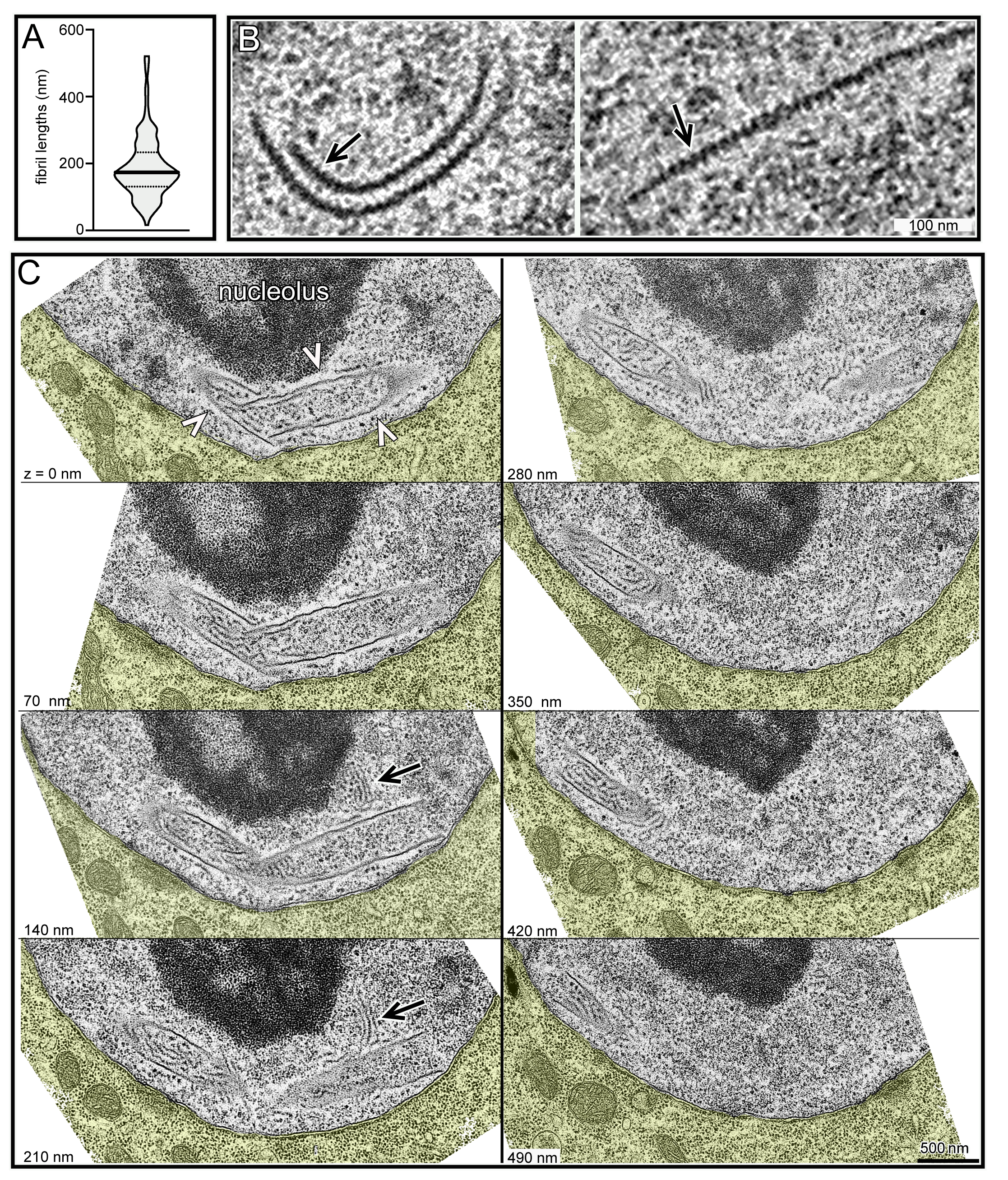

Supplement: S9 Fig — A. Measurements of individual fibril lengths (n = 150) taken from TEM micrographs; graphing with GraphPad Prism software version 9.5. B. High magnification of three long fibrils (344, 477, and 497 nm); sample as in Fig 7D. Note the numerous striations (arrows) which are approximately orthogonal to the long axis of the fibril. C. Images of sequential, 70 nm thick Z-sections through a curved CERV rod. The perimeter of the rod has an electron-lucent margin (white arrowheads), and electron-dense fibrils are near the perimeter and inside the rod. The arrows at 140nm and 210nm indicate a few electron-dense fibrils which are associated with the nucleolus, but do not appear to have become incorporated into the rod. (TIF) [file pgen.1010804.s016.tif]
